# Mitochondrial dysfunction activates ADAMTS-5 expression via mt-dsRNA-PKR-Spi-1 axis in osteoarthritic chondrocytes

**DOI:** 10.1016/j.isci.2026.115980

**Published:** 2026-05-20

**Authors:** Yulong Mu, Shuaichen Yan, Yizhe Wang, Boning Liang, Rui Tang, Huapu Yang, Liang Ma, Yuankai Zhang, Deqiang Li

**Affiliations:** 1 Department of Orthopedics, Qilu Hospital of Shandong University, 107 Wenhuaxi Road, Jinan 250012, China

**Keywords:** Biological Sciences

## Abstract

Osteoarthritis (OA) involves cartilage degradation by ADAMTS-5, but how ADAMTS-5 expression is activated remains unclear. A recent study found that mitochondrial double-strand RNA (mt-dsRNA) and its leakage into the cytosol activate protein kinase R (PKR), which facilitates OA progression. However, the mechanisms are yet to be clarified. In this study, we focused on how mt-dsRNAs and PKR signaling activate ADAMTS-5 expression in chondrocytes undergoing mitochondrial dysfunction. Our findings revealed that mitochondrial dysfunction triggered the leakage of mt-dsRNAs into the cytosol. These mt-dsRNAs activated PKR to initiate Spi-1-dependent ADAMTS-5 transcription. Increased levels of mt-dsRNAs and PKR activation in the cartilage from OA patients and mice corroborate our findings. More importantly, PKR conditional knockout from articular chondrocytes alleviates the pathological phenotypes of OA mice. Our research offers a deep understanding of OA driven by mitochondrial dysfunction in chondrocytes and proposes mt-dsRNAs-PKR as potential targets for OA drugs.

## Highlights

• Mitochondrial dysfunction induces mt-dsRNA efflux into the cytosol of OA chondrocytes
• Cytosolic mt-dsRNAs activate PKR signaling, driving ADAMTS-5 expression via Spi-1
• Chondrocyte-specific PKR knockout attenuates cartilage degeneration in OA mice
• Targeting the mt-dsRNA-PKR axis may offer a novel therapeutic strategy for OA

## Graphical abstract

**Figure:**
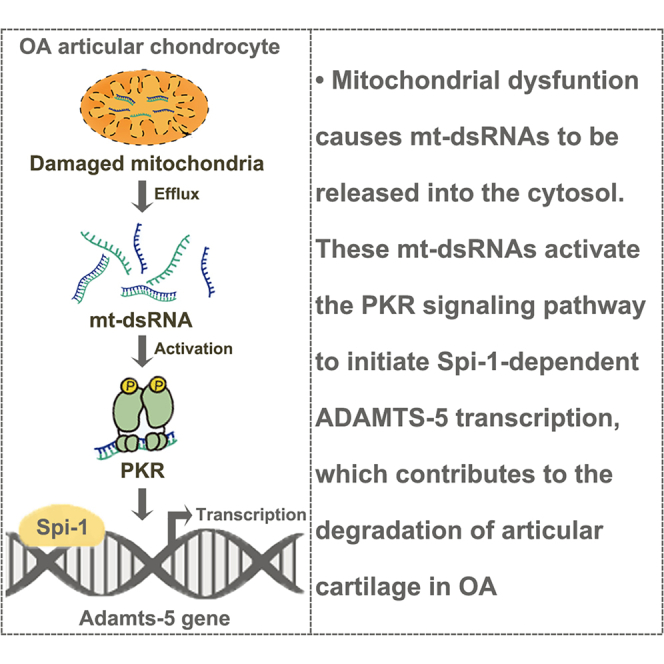

## Introduction

Osteoarthritis (OA) is a common source of disability among older adults.^1^ The deterioration of articular cartilage (AC) leads to joint pain and impaired function.^2^ While factors such as aging, obesity, and excessive joint stress are known risks, OA is increasingly understood as more complex than mere “wear and tear.”^3^^,^^4^ A key factor in the development of OA is the disruption of the balance between anabolic and catabolic processes that sustain the extracellular matrix.^5^^,^^6^ These metabolic changes are characterized by a decrease in anabolic gene expression and an increase in catabolic gene expression.^7^

A disintegrin and metalloproteinase with thrombospondin motifs-5 (ADAMTS-5) is an essential zinc-dependent endopeptidase that plays a vital role in ensuring the equilibrium and stability of tissues and organs.^8^ This enzyme is extensively present throughout the musculoskeletal system and is particularly activated by inflammatory cytokines in specific areas of knee joint explants.^9^ ADAMTS-5 is responsible for the breakdown of aggrecan in the cartilage of mice and bovine.^10^^,^^11^^,^^12^ In human OA cartilage, the reduction of aggrecan loss was achieved through the suppression of ADAMTS-5 using siRNA.^13^ In mice, the *in vivo* deletion of ADAMTS-5 led to a reduction in mechanical allodynia, prevention of cartilage degradation, and decreased thickening of subchondral bone.^14^^,^^15^^,^^16^^,^^17^ The signaling pathways and key factors that regulate ADAMTS-5 activation during OA development have been extensively investigated.

Our team previously published two studies that proposed a mechanistic framework where ADAMTS-5 expression activation in OA is controlled by the interplay of DNA methylation, histone H3K9 dimethylation, and the transcription factor Spi-1. In healthy chondrocytes, ADAMTS-5 expression is suppressed by compact chromatin structures created by DNA methylation and H3K9 dimethylation. In contrast, in OA chondrocytes, DNA demethylation interferes with the attachment of methyl-CpG binding protein 2 to the ADAMTS-5 promoter. Lysine-specific demethylase 1 prevents the dimethylation of histone H3K9. The combination of decreased histone H3K9 dimethylation and DNA demethylation leads to a relaxation of chromatin structure, allowing the transcription factor Spi-1 to bind to the ADAMTS-5 promoter and trigger the activation of ADAMTS-5 expression.^18^^,^^19^ Nevertheless, the upstream biological processes that regulate Spi-1-dependent ADAMTS-5 expression in OA are still unclear.

Mitochondria are essential in aging. These subcellular organelles are primarily responsible for energy production through oxidative phosphorylation. However, as individuals age, mitochondrial function deteriorates. This reduction is seen as a “hallmark of aging” and plays a role in the development of age-related conditions like OA.^20^ When mitochondria are not functioning properly, there is an increase in reactive oxygen species (ROS), which intensifies oxidative stress, decreases ATP production, reduces the ability to synthesize matrix, and elevates apoptosis in OA chondrocytes.^20^^,^^21^ In aged and OA cartilage, mitochondrial dysfunction activates the JNK-MAPK/cFos/AP1 pathway via ROS, prompting the production of matrix-degrading enzymes and the expression of pro-inflammatory genes.^22^ The regulation and continuous renewal of mitochondrial double-stranded RNAs (mt-dsRNAs) are essential for determining cell fate. Heightened oxidative stress not only increases the generation of mt-dsRNAs but also leads to their leakage from the mitochondria into the cytoplasm.^23^ In the cytoplasm, these mt-dsRNAs can be recognized by various RNA sensor proteins.^24^^,^^25^^,^^26^ protein kinase R (PKR) is one such sensor that can subsequently activate eukaryotic initiation factor 2α (eIF2α) at the Ser51 site.^24^^,^^27^^,^^28^ A previous study showed that mt-dsRNA expression and its release into the cytosol were enhanced in chondrocytes under OA-inducing conditions, leading to PKR activation and promoting OA development.^29^ However, the mechanisms underlying that mt-dsRNA-activated PKR contributes to OA development remain unclear.

In this research, we explored mt-dsRNAs as a crucial element in mitochondrial dysfunction within the chondrocyte and examined their potential role in the OA pathophysiology. Our focus was on the involvement of mt-dsRNAs and the PKR signaling in triggering ADAMTS-5 expression when chondrocytes undergo mitochondrial dysfunction. Notably, we confirmed our results using damaged cartilage from OA patients and cartilage from murine OA models. Finally, we evaluated OA phenotype of mice with PKR conditional knockout from articular chondrocytes. Overall, the data from current study identify mt-dsRNAs as intracellular dsRNAs that serve pathophysiological functions linking mitochondria and OA.

## Results

### Mitochondrial dysfunction activates ADAMTS-5 expression in chondrocytes

**Figure 1:**
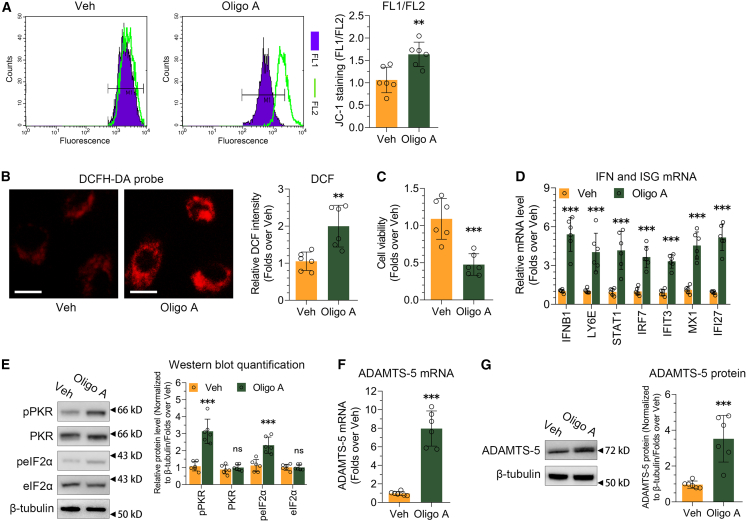
Mitochondrial dysfunction activates ADAMTS-5 expression in chondrocytes (A–D) The impact of inhibiting mitochondrial respiratory chain with oligo A (30 μg/mL) on mitochondrial membrane potential (A, *n* = 6), ROS generation (B, *n* = 6), cell survival (C, *n* = 6), and the mRNA expression of IFNB1 and ISGs (D, *n* = 6) in the SW1353 cell line. Scale bars in (B) are 20 μm. (E) Immunoblotting was used to analyze the phosphorylation of PKR and eIF2α following oligo A treatment in the SW1353 cell line, with quantification displayed on the right (*n* = 6). (F and G) RT-qPCR and immunoblotting were conducted to assess the expression of ADAMTS-5 mRNA (F, *n* = 6) and protein (G) in response to oligo A treatment in the SW1353 cell line, with immunoblotting quantification shown on the right (*n* = 6). Data (mean ± std) was representative of three independent experiments. An unpaired student’s *t* test was conducted to compare two groups. ∗, *p* < 0.05, ∗, *p* < 0.01, ∗∗∗, *p* < 0.001; ns, no significance.

Mitochondrial dysfunction results in elevated ROS levels, which trigger inflammation and diminish the biosynthetic ability of chondrocytes.^30^^,^^31^ Our research aimed to explore how mitochondrial dysfunction might trigger ADAMTS-5 expression and result in chondrocyte deterioration in OA. To induce mitochondrial dysfunction, we exposed the SW1353 cell line to oligo A, which inhibits ATP synthase in the mitochondrial respiratory chain. Oligo A disrupted the mitochondrial membrane potential (Figure 1A) and elevated the ROS response (Figure 1B). Oligo A led to the attenuation of cell viability (Figure 1C) and the induction of interferon β (IFN-β) and IFN-stimulated genes (ISGs) (Figure 1D). Oligo A increased the phosphorylation of PKR and its downstream target eIF2α (Figure 1E). To rule out effects specific to oligo A, SW1353 was exposed to FCCP, an uncoupler of mitochondrial oxidative phosphorylation. FCCP-induced mitochondrial dysfunction also resulted in an increased ROS response (Figure S1A) and cell viability attenuation (Figure S1B). PKR was activated and ISGs expression was induced by FCCP (Figures S1C and S1D). We confirmed our findings using the human chondrocyte cell line CHON-001. Oligo A disrupted the mitochondrial membrane potential (Figure S2A), increased the ROS response (Figure S2B), and lowered cell viability (Figure S2C). In CHON-001 cells, oligo A also activated the PKR signaling pathway (Figure S2D) and ISGs expression (Figure S2E).

To assess how mitochondrial dysfunction impacts crucial genes like MMPs and ADAMTSs, which are involved in the AC degradation in OA, we examined the expression levels of MMP-1, MMP-3, MMP-9, MMP-13, ADAMTS-4, and ADAMTS-5 following oligo A treatment in SW1353 cell line. The mRNA and protein levels of MMP-1, MMP-3, MMP-9, MMP-13, and ADAMTS-4 remained unchanged with oligo A treatment (Figures S1E and S1F). In contrast, there was a significant increase in the mRNA and protein expressions of ADAMTS-5 in response to oligo A treatment (Figures 1F and 1G). Similarly, FCCP treatment also led to a significant up-regulation of ADAMTS-5 mRNA and protein expressions in SW1353 cell line (Figures S1G and S1H). Oligo A significantly up-regulated the mRNA and protein expressions of ADAMTS-5 in CHON-001 cell line (Figures S2F and S2G).

### PKR is involved in the ADAMTS-5 expression activated by mitochondrial dysfunction in chondrocytes

After observing that mitochondrial dysfunction led to the activation of PKR signaling and ADAMTS-5 expression in chondrocytes, we hypothesized that the activation of ADAMTS-5 expression was mediated by PKR signaling. We used siRNA to downregulate ADAMTS-5 expression in SW1353 cell line (Figures S3A and S3B). The results showed no differences in the phosphorylation levels of PKR and its downstream target eIF2α between scramble control and ADAMTS-5 siRNA group (Figure S3C). The mRNA expressions of ISGs were unaffected by ADAMTS-5 knockdown (Figure S3D), indicating that ADAMTS-5 does not act upstream of PKR.

**Figure 2:**
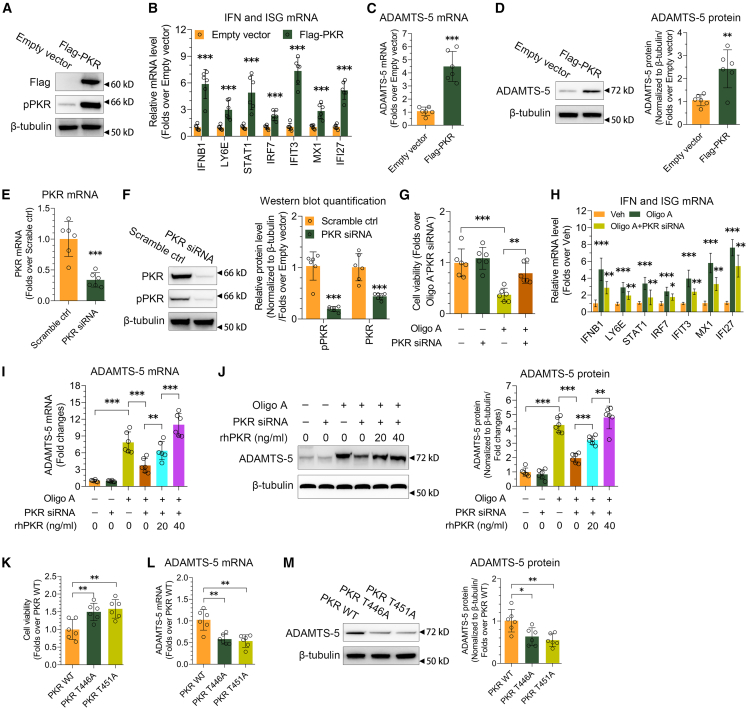
Mitochondrial dysfunction activates ADAMTS-5 expression via PKR in chondrocytes (A) Analysis of PKR overexpression introduced via a lentivirus vector carrying the PKR coding sequence in the SW1353 cell line using immunoblotting. (B) Examination of IFNB1 and ISG mRNA levels following PKR overexpression (*n* = 6). (C and D) Evaluation of ADAMTS-5 mRNA (C, *n* = 6) and protein (D) levels in response to PKR overexpression in the SW1353 cell line through RT-qPCR and immunoblotting. The quantification of immunoblotting results is displayed on the right (*n* = 6). (E and F) Analysis of PKR mRNA (E, *n* = 6) and protein (F) levels following siRNA-mediated PKR knockdown in the SW1353 cell line using RT-qPCR and immunoblotting. The quantification of immunoblotting results is shown on the right (*n* = 6). (G and H) Assessment of cell viability (G, *n* = 6), and IFNB1 and ISG mRNA levels (H, *n* = 6) in response to oligo A treatment and/or PKR knockdown in the SW1353 cell line. (I and J) Evaluation of ADAMTS-5 mRNA (I, *n* = 6) and protein (J) levels in response to oligo A treatment, PKR knockdown and/or recombinant human PKR protein treatment in the SW1353 cell line using RT-qPCR and immunoblotting. The quantification of immunoblotting results is displayed on the right (*n* = 6). (K) Impact of mutating one of the two critical phosphorylation residues (T446A or T451A) of PKR on cell viability (*n* = 6). (L and M) Analysis of ADAMTS-5 mRNA (L, *n* = 6) and protein (M) levels in response to the mutation of one of the two key phosphorylation residues (T446A or T451A) of PKR in the SW1353 cell line using RT-qPCR and immunoblotting. The quantification of immunoblotting results is shown on the right (*n* = 6). Data (mean ± std) was representative of three independent experiments. To compare two groups, an unpaired student’s *t* test was conducted in (B, C, D, E, F, and H). For analyses involving multiple groups, one-way (K, L, and M) and two-way (G, I, and J) ANOVA were employed. ∗, *p* < 0.05, ∗, *p* < 0.01, ∗∗∗, *p* < 0.001; ns, no significance.

When PKR was overexpressed in SW1353 cell line using a lentivirus vector (Figure 2A), there was a notable increase in the expression levels of ADAMTS-5 and ISGs (Figures 2B–2D). We utilized siRNA to reduce PKR expression in SW1353 cell line (Figures 2E and 2F). The cell viability attenuation caused by oligo A was significantly mitigated following PKR knockdown (Figure 2G). The activation of ISGs by oligo A was diminished to varying extents due to PKR knockdown (Figure 2H). ADAMTS-5 expression activation by oligo A was blunted significantly in response to PKR knockdown (Figures 2I and 2J). More importantly, when recombinant PKR protein was added to the cell culture to recover the function of PKR abated by siRNA (Figure S3E), ADAMTS-5 expression was re-activated (Figures 2I and 2J), suggesting that PKR played an important role in ADAMTS-5 expression activated by mitochondrial dysfunction.

In the mutagenesis experiment, we engineered PKR mutants at two essential phosphorylation sites, Thr446 and Thr451, to identify the critical residue necessary for PKR function in chondrocytes undergoing mitochondrial dysfunction. Cell viability improved with PKR T446A or PKR T451A compared to PKR WT (Figure 2K). The expression of ADAMTS-5 was significantly reduced by PKR T446A or PKR T451A compared to PKR WT (Figures 2L and 2M), indicating that both phosphorylation sites are critical for the activation of ADAMTS-5 expression via PKR in the context of mitochondrial dysfunction.

### Mitochondrial dysfunction leads to the release of mt-dsRNAs into the cytosol to activate PKR

**Figure 3:**
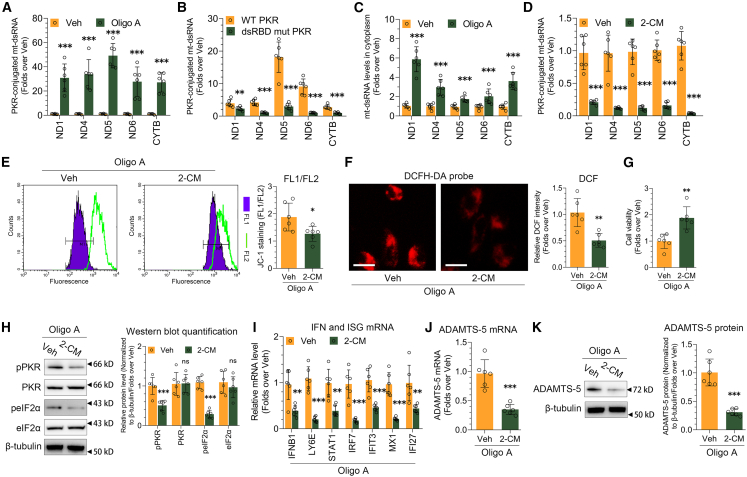
Mitochondrial dysfunction induces the cytosolic efflux of mt-dsRNAs to activate PKR (A) The interaction between PKR and mtRNA following oligo A treatment was assessed using PKR fCLIP-qPCR analysis (*n* = 6). (B) The interaction of PKR with mtRNA was analyzed in the presence of wild-type (WT) and dsRBD-mut PKR through fCLIP-qPCR analysis (*n* = 6). (C) The levels of mtRNA in the cytoplasm of the SW1353 cell line were measured in response to oligo A treatment using RT-qPCR (*n* = 6). (D) The interaction between PKR and mtRNA after 2-CM (50 μM) treatment was evaluated using PKR fCLIP-qPCR analysis (*n* = 6). (E–I) The mitochondrial membrane potential (E, *n* = 6), ROS production (F, *n* = 6), cell viability (G, *n* = 6), phosphorylation of PKR and eIF2α (H), and the expression of IFNB1 and ISGs mRNA (I, *n* = 6) were examined in response to oligo A and 2-CM treatment in the SW1353 cell line. The scale bars in (F) are 20 μm. The quantification of the western blot is displayed to the right of the blot images (*n* = 6). (J and K) RT-qPCR and immunoblotting were used to analyze the expression of ADAMTS-5 mRNA (J, *n* = 6) and protein (K) in response to oligo A and 2-CM treatment in the SW1353 cell line. The quantification of immunoblotting is shown on the right (*n* = 6). Data (mean ± std) was representative of three independent experiments. An unpaired student’s *t* test was conducted to compare two groups. ∗, *p* < 0.05, ∗, *p* < 0.01, ∗∗∗, *p* < 0.001; ns, no significance.

We then aimed to identify how PKR was activated during mitochondrial dysfunction. TNF-α and IL-1β emerged as two possible candidates, being significant inflammatory factors in OA^32^ and known to be inducible during immune responses, with a strong association with PKR.^33^^,^^34^ We assessed the secretion of TNF-α and IL-1β from SW1353 cells using ELISA, but observed no change in their levels following oligo A treatment (Figure S4A). In another approach, we investigated the role of mt-dsRNAs, an essential type of endogenous dsRNAs, in the activation of PKR when mitochondrial dysfunction is induced. Using formaldehyde, we crosslinked PKR-RNA complexes and found that oligo A increased the interaction between PKR and mtRNA (Figure 3A). To evaluate the specificity of the interaction, we engineered a mutant PKR with decreased ability to bind dsRNA (dsRBD-mut), which showed a notably reduced affinity for mtRNAs compared to the wild-type PKR (Figure 3B).

Considering that PKR is largely located in the cytosol, we explored whether oligo A would result in a heightened release of mt-dsRNAs into the cytosol. One reason could be the lowered levels of polynucleotide phosphorylase (PNPase), potentially leading to the escape of mt-dsRNA through the mitochondrial membrane.^35^ We assessed PNPase expression following oligo A treatment and observed a slight, though not statistically significant, increase in its mRNA levels (Figure S4B). PNPase protein levels remained stable in both total cell lysates and isolated mitochondria when treated with oligo A, as shown in Figure S4C.

Another potential explanation for the increased release of mt-dsRNAs into the cytosol might be a disturbance in the mitochondrial membrane potential. We separated the free cytosolic compartment from the organelles (membrane fraction) and the nucleus, followed by RNA extraction from these isolated compartments. The effectiveness of this fractionation was validated by assessing various marker proteins through western blot analysis (Figure S4D). Our findings indicated a significant rise in cytosolic mtRNA levels following oligo A treatment (Figure 3C). We also verified that oligo A resulted in heightened levels of cytosolic mtRNA in CHON-001 cell line (Figures S4E–S4G).

In addition to mt-dsRNAs, human cells generate a range of noncoding and repeat RNAs that can trigger PKR activation.^36^ We investigated whether mt-dsRNAs significantly contribute to PKR activation when mitochondrial dysfunction is induced. Cells underwent treatment with 2′-C-methyladenosine (2-CM), a small-molecule inhibitor that specifically targets mitochondrial RNA polymerase. This intervention led to an 80%–90% reduction in the interaction between PKR and mtRNA, as depicted in Figure 3D. This reduction was also confirmed in CHON-001 cell line following 2-CM treatment (Figure S4H). We found that the reduction in mitochondrial membrane potential induced by oligo A was mitigated when treated with 2-CM (Figure 3E). 2-CM reduced oxidative stress induced by oligo A (Figure 3F), leading to a notable improvement in cell viability (Figure 3G). In the absence of mtRNA, oligo A failed to trigger the activation of PKR and eIF2α, resulting in a decreased level of ISG (Figures 3H and 3I). The expression of ADAMTS-5 triggered by oligo A was significantly reduced in response to 2-CM treatment (Figures 3J and 3K).

### PKR activated by mt-dsRNAs enhances the accumulation of Spi-1 in the nuclear and the enrichment of Spi-1 on ADAMTS-5 promoter

PKR activation is inherently linked to caspase-dependent cell death.^37^ We wonder if caspase-dependent cell death is involved in the ADAMTS-5 activation by PKR. Pan-caspase inhibitor Z-VAD-FMK was employed to inhibit caspases functions. We found that Z-VAD-FMK failed to change the ADAMTS-5 mRNA and protein expression activation by oligo A (Figures S5A and S5B). Also, ADAMTS-5 expression activation by exogenous PKR overexpression remained unchanged between with and without Z-VAD-FMK treatment (Figures S5C and S5D), suggesting that caspase-mediated cell death is not involved in the activation of ADAMTS-5 expression by PKR.

**Figure 4:**
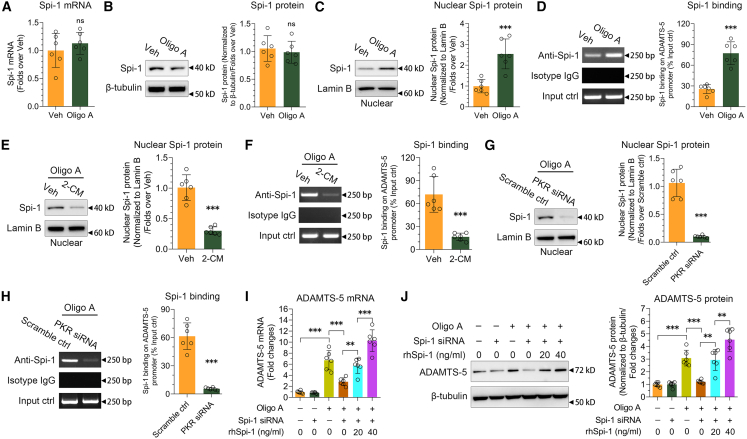
PKR activated by mt-dsRNAs enhances the translocation of Spi-1 to nuclear and its binding on ADAMTS-5 promoter (A and B) The expression of Spi-1 mRNA (A, *n* = 6) and protein (B) in the SW1353 cell line was analyzed using RT-qPCR and immunoblotting following oligo A treatment. The quantification of the immunoblotting results is displayed on the right (*n* = 6). (C) Immunoblotting was used to assess Spi-1 protein levels in the nucleus of the SW1353 cell line after oligo A treatment, with quantification shown on the right (*n* = 6). (D) The binding of Spi-1 to the ADAMTS-5 promoter in response to oligo A treatment was evaluated using ChIP, with quantification on the right (*n* = 6). (E) The nuclear expression of Spi-1 protein in the SW1353 cell line was examined through immunoblotting after treatment with oligo A and 2-CM, with quantification on the right (*n* = 6). (F) ChIP was used to investigate Spi-1 binding to the ADAMTS-5 promoter following oligo A and 2-CM treatment, with quantification on the right (*n* = 6). (G) Immunoblotting analysis was conducted to determine Spi-1 protein levels in the nucleus of the SW1353 cell line after oligo A treatment and PKR knockdown, with quantification on the right (*n* = 6). (H) ChIP analysis was performed to assess Spi-1 binding to the ADAMTS-5 promoter in response to oligo A treatment and PKR knockdown, with quantification on the right (*n* = 6). (I and J) The expression of ADAMTS-5 mRNA (I, *n* = 6) and protein (J) in the SW1353 cell line was analyzed using RT-qPCR and immunoblotting following oligo A treatment, Spi-1 knockdown and/or recombinant human Spi-1 treatment, with quantification on the right (*n* = 6). Data (mean ± std) was representative of three independent experiments. To compare two groups, an unpaired Student’s *t* test was conducted in (A–H). For analyses involving multiple groups, two-way (I and J) ANOVA were employed. ∗, *p* < 0.05, ∗, *p* < 0.01, ∗∗∗, *p* < 0.001; ns, no significance.

In our earlier reports,^18^^,^^19^ we have identified that the transcription factor Spi-1 is involved in the activation of ADAMTS-5 expression within AC by binding to the ADAMTS-5 promoter in OA. After observing that mitochondrial dysfunction activated ADAMTS-5 expression through PKR signaling, we sought to assess the impact of mitochondrial dysfunction and PKR signaling on Spi-1. Oligo A did not alter the mRNA and protein levels of Spi-1 in SW1353 cell line (Figures 4A and 4B). When nuclear proteins were extracted, western blot revealed a significant increase in Spi-1 levels in the nucleus following oligo A treatment compared to the vehicle control (Figure 4C). ChIP data showed an enrichment of Spi-1 on the ADAMTS-5 promoter in response to oligo A treatment (Figure 4D).

Upon using 2-CM to suppress mt-dsRNAs expression, the accumulation of Spi-1 in the nucleus following oligo A treatment was hindered (Figure 4E), leading to a notable reduction in Spi-1 binding to the ADAMTS-5 promoter (Figure 4F). When siRNA was utilized to reduce PKR expression, the accumulation of Spi-1 in the nucleus after oligo A treatment was obstructed (Figure 4G), resulting in a significant decrease in Spi-1 binding on the ADAMTS-5 promoter (Figure 4H).

siRNA was applied to knock down Spi-1 in SW1353 cell line (Figures S6A and S6B), revealing that oligo A-induced ADAMTS-5 expression was diminished due to Spi-1 knockdown (Figures 4I and 4J). More importantly, when recombinant Spi-1 protein was added to the cell culture to recover the function of Spi-1 abated by siRNA (Figure S6C), ADAMTS-5 expression was re-activated (Figures 4I and 4J), suggesting that Spi-1 played an essential role in ADAMTS-5 expression. Beyond SW1353 cell line, mt-dsRNAs and PKR signaling, in the mitochondrial dysfunction, prompted Spi-1 nuclear accumulation and enrichment on the ADAMTS-5 promoter in CHON-001 cell line (Figures S6D–S6K). These findings indicate that the mt-dsRNA-PKR-Spi-1 pathway is crucial for the activation of ADAMTS-5 expression in OA chondrocytes.

### mt-dsRNAs expression up-regulation and PKR activation in the articular cartilage of OA mice and OA patients

**Figure 5:**
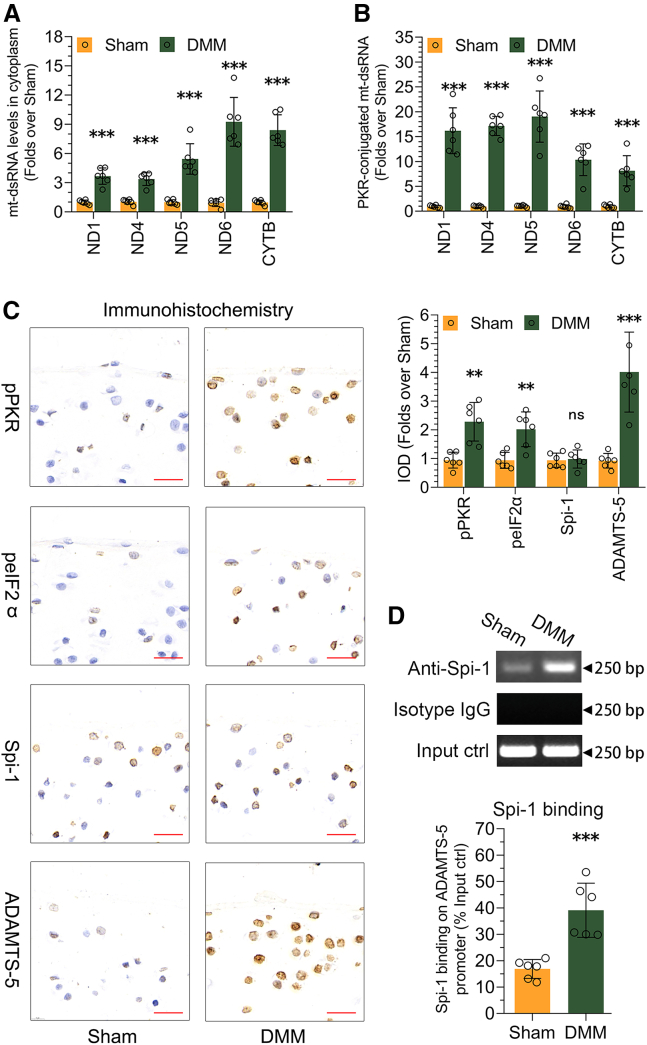
mt-dsRNAs expression up-regulation and PKR activation in damaged cartilage of OA mice (A) The levels of mtRNA in the cytoplasm of chondrocytes from the AC of Sham and DMM mice were assessed using RT-qPCR (*n* = 6). (B) The interaction between PKR and mtRNA in chondrocytes from the AC of Sham and DMM mice was analyzed through PKR fCLIP-qPCR (*n* = 6). (C) Immunohistochemistry was used to analyze the expression of pPKR, peIF2α, Spi-1, and ADAMTS-5 proteins in the AC of Sham and DMM mice, with the quantification of integrated optical density (IOD) presented on the right (*n* = 6). (D) The binding of Spi-1 to the ADAMTS-5 promoter in the AC of Sham and DMM mice was examined using ChIP, with quantification displayed below the electrophoretic gel image (*n* = 6). Data (mean ± std) was representative of three independent experiments. An unpaired student’s *t* test was conducted to compare two groups. ∗, *p* < 0.05, ∗, *p* < 0.01, ∗∗∗, *p* < 0.001; ns, no significance.

Chondrocyte impairment is a significant marker of OA.^5^ After gathering data from *in vitro* cell cultures, we aimed to translate our findings to *in vivo* settings. We established an OA mouse model through DMM surgery (Figures S7A–S7E), harvested chondrocytes from the AC of both Sham and DMM groups, and assessed the mtRNA expression within the cytoplasm. The results showed an elevated expression of mtRNAs in the cytoplasm of chondrocytes from DMM mice compared to Sham mice (Figure 5A), which was accompanied by an increased PKR-mtRNA interaction in the chondrocytes from DMM mice compared to those from Sham mice (Figure 5B). We then examined whether the elevation of mtRNAs in DMM mice initiated PKR signaling and the expression of Spi-1 and ADAMTS-5. We observed a heightened signal for pPKR, peIF2α, and ADAMTS-5 in DMM cartilage compared to Sham cartilage (Figure 5C). While Spi-1 expression remained unchanged between DMM and Sham mice (Figure 5C), there was an enrichment of Spi-1 on the ADAMTS-5 promoter in DMM mice compared to Sham mice, aligning with our *in vitro* data. To rule out DMM-specific effects, we developed another animal model, the ACLT-induced OA mouse model (Figures S7F–S7J), where we examined mt-dsRNA expression, PKR-mt-dsRNA interaction, the expression of pPKR, peIF2α, Spi-1, and ADAMTS-5, and the binding of Spi-1 on the ADAMTS-5 promoter. The ACLT-induced OA model exhibited a pattern similar to the DMM-induced OA model (Figure S8).

**Figure 6:**
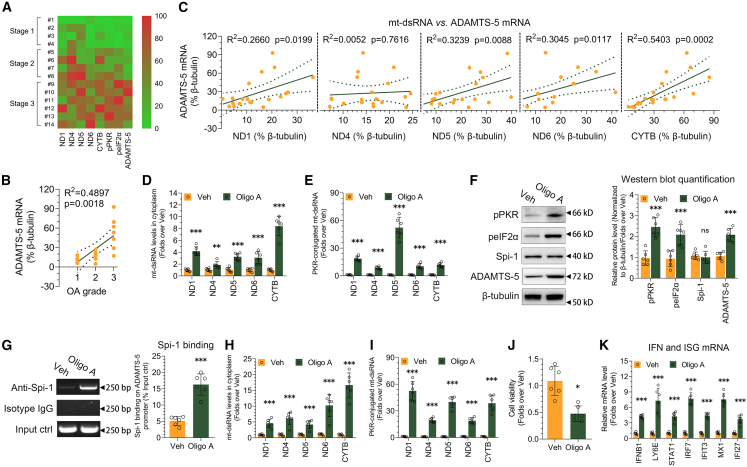
mt-dsRNAs expression up-regulation and PKR activation in damaged cartilage of OA patients (A) Heatmap of mt-dsRNA expression levels, PKR signaling pathway, and ADAMTS-5 mRNA expression in the AC of 14 OA patients in different OA stage according to Outerbridge scale. (B) The relationship between ADAMTS-5 mRNA expression and the severity of OA in the AC of OA patients via Spearman’s method. (C) The association between ADAMTS-5 mRNA expression and mt-dsRNAs levels in the AC of OA patients. (D) The impact of oligo A treatment on mtRNA levels in the AC of OA patients, assessed using RT-qPCR (*n* = 6). (E) The interaction between PKR and mtRNA in the AC of OA patients following oligo A treatment, analyzed through PKR fCLIP-qPCR (*n* = 6). (F) Immunoblotting analysis of pPKR, peIF2α, Spi-1, and ADAMTS-5 protein levels in the AC of OA patients in response to oligo A treatment, with quantification displayed on the right (*n* = 6). (G) Spi-1 binding to the ADAMTS-5 promoter in the AC of OA patients after oligo A treatment, examined by ChIP, with quantification shown on the right (*n* = 6). (H) The effect of oligo A treatment on mtRNA levels in the primary chondrocytes of OA patients, evaluated using RT-qPCR (*n* = 6). (I) The interaction between PKR and mtRNA in the primary chondrocytes of OA patients upon oligo A treatment, assessed by PKR fCLIP-qPCR (*n* = 6). (J and K) The impact of mitochondrial respiratory chain inhibition by oligo A on cell viability (J, *n* = 6), and the expression of IFNB1 and ISG mRNA (K, *n* = 6) in the primary chondrocytes of OA patients. Data (mean ± std) was representative of three independent experiments. An unpaired Student’s *t* test was conducted to compare two groups. ∗, *p* < 0.05, ∗, *p* < 0.01, ∗∗∗, *p* < 0.001; ns, no significance.

To expand our results and assess their clinical significance, we conducted an analysis of human cartilage obtained from patients with OA. From 14 OA patients undergoing TKR, femoral cartilage lesions were obtained and clinically evaluated by experienced surgeon according to the modified Outerbridge scale.^38^ Femoral cartilage were harvested from chondral lesions, graded 1 (mild damage) to 3 (severe damage). RNA and protein were isolated from the cartilage. RT-PCR and immunoblotting were employed to investigate the expression levels of mt-dsRNAs, phosphorylated PKR and eIF2α, and ADAMTS-5. We are unable to compare the data of OA cartilage with that of normal cartilage because normal cartilage sampling may be unethical or is often impractical in subjects with normal joints. Instead, we used OA patients in stage 1 as baseline. The expression of mt-dsRNAs was up-regulated significantly, especially ND1, ND4, ND6 and CYTB, as the severity of OA increased (Figure 6A and Table S2). The positive relation of phosphorylated PKR and eIF2α, and ADAMTS-5 expression with OA severity was observed (Figure 6A and Table S2). Further, we assessed the expression of ADAMTS-5 in relation to the extent of cartilage damage in these patients. A significant positive correlation was found between ADAMTS-5 expression and the severity of OA (Figure 6B). Subsequently, we explored the connection between ADAMTS-5 and mt-dsRNAs expression in the cartilage of OA patients. Except for ND4, there was a notable positive correlation between the expression levels of ADAMTS-5 and mt-dsRNAs (Figure 6C).

To set up for tissue culture, we carefully dissected AC tissues into smaller fragments. Following this, we induced mitochondrial dysfunction by introducing oligo A. In line with the results from *in vitro* cell line, we observed a significant rise in cytosolic mtRNAs levels following oligo A treatment (Figure 6D), and the interaction between PKR and mtRNA was intensified by oligo A (Figure 6E). Oligo A treatment led to increased protein expressions of pPKR, peIF2α, and ADAMTS-5 in human cartilage (Figure 6F). Although Spi-1 expression did not change in human AC with or without oligo A treatment (Figure 6F), there was an observed enrichment of Spi-1 on the ADAMTS-5 promoter in human cartilage treated with oligo A compared to the vehicle control (Figure 6G).

We performed further evaluations utilizing primary human chondrocytes obtained from the cartilage of OA patients. When these primary chondrocytes were treated with oligo A, there was an up-regulation in the expression of mt-dsRNAs (Figure 6H) and an enhanced interaction between PKR and mtRNAs (Figure 6I). Moreover, oligo A resulted in cell viability attenuation and an elevation in the mRNA expression of ISGs (Figures 6J and 6K).

### Alleviation of pathological phenotypes of OA mice in response to PKR conditional knockout from articular chondrocytes

**Figure 7:**
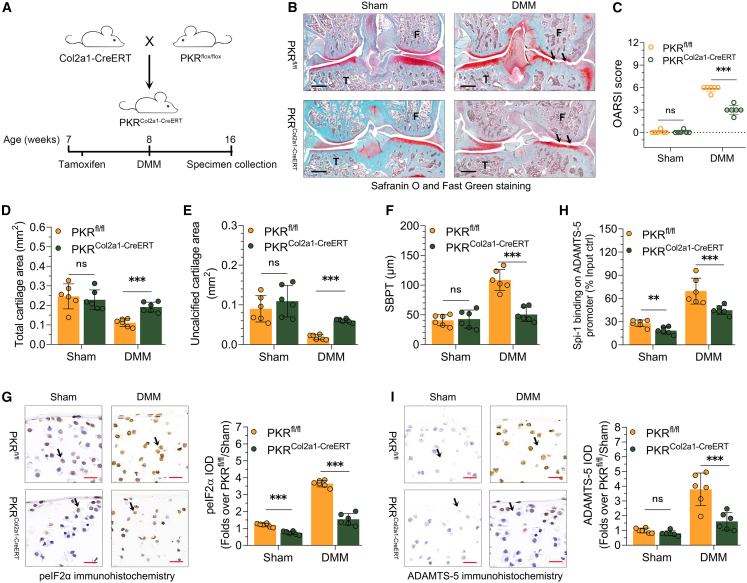
The conditional deletion of PKR from articular chondrocytes alleviates the pathological characteristics of mice with OA (A) Establishment of mice model with PKR conditional knockout from articular chondrocytes. (B) Representative photomicrographs of knee joint sections obtained from PKR^fl/fl^ and PKR^Col2a1-CreERT^ mice undergoing Sham or DMM surgery, stained with Safranin O/fast green. The arrows represent cartilage damage. The scale bars are 100 μm. (C) OARSI score quantification of the severity of OA in mice, as shown in Figure 7A; *n* = 6 per group. (D) The total cartilage area as quantified in Figure 7A; *n* = 6 per group. (E) Uncalcified cartilage area quantification as shown in Figure 7A; *n* = 6 per group. (F) Subchondral bone plat thickness quantification for mice depicted in Figure 7A; *n* = 6 per group. (G) Representative immunohistochemistry staining and quantification of IOD of phosphorylated eIF2α protein from knee joint sections of PKR^fl/fl^ and PKR^Col2a1-CreERT^ mice undergoing DMM or Sham surgery. The arrows point at the positively stained chondrocytes. *n* = 6 per group. (H) Spi-1 bindings on mouse ADAMTS-5 promoter in AC of PKR^fl/fl^ and PKR^Col2a1-CreERT^ mice subjected to Sham or DMM surgery determined by ChIP; *n* = 6 per group. (I) Representative immunohistochemistry staining and quantification of IOD of phosphorylated eIF2α protein from knee joint sections of PKR^fl/fl^ and PKR^Col2a1-CreERT^ mice undergoing DMM or Sham surgery. The arrows point at the positively stained chondrocytes. *n* = 6 per group. Data (mean ± std) was representative of three independent experiments. For analyses involving multiple groups, two-way ANOVA were employed. ∗, *p* < 0.05, ∗∗, *p* < 0.01, ∗∗∗, *p* < 0.001; ns, no significance.

In order to evaluate the functional significance of mt-dsRNA-activated PKR signaling in ADAMTS-5 expression activation in OA AC, we generated chondrocyte-conditioned knockout PKR (PKR^Col2a1-CreERT^) mice (Figures 7A and S9). On the knee joints of PKR^Col2a1-CreERT^ and PKR^fl/fl^ mice, DMM surgery was performed. Knee joints from mice were harvested and stained with Safranin O/fast green at week 8 following surgery. With a high OARSI score, the DMM PKR^fl/fl^ mice exhibited degenerative cartilage damage including cartilage erosion and loss (Figures 7B and 7C). On the other hand, compared to PKR^fl/fl^ mice, DMM mice with PKR conditional knockout from articular chondrocytes exhibited less severe injury to AC (Figure 7B) and a lower OARSI score (Figure 7C). Furthermore, it was observed that DMM mice harboring PKR conditional knockout from articular chondrocytes exhibited augmented total cartilage area (Figure 7D) and uncalcified cartilage area (Figure 7E) in the proximal tibia. The subchondral bone plate thickness adjacent to destroyed cartilage in the proximal tibia was less in PKR^Col2a1-CreERT^ mice than that of PKR^fl/fl^ mice (Figure 7F). These results indicate that the conditional knockout of PKR from articular chondrocytes ameliorated the cartilage degeneration associated with OA.

Tissue sections were prepared from knee joints of PKR^fl/fl^ mice and PKR^Col2a1-CreERT^ mice subjected to Sham or DMM surgery. Immunohistochemistry assay was performed to evaluate phosphorylated eIF2α protein expression in the AC. PKR conditional knockout from articular chondrocytes decreased the staining of phosphorylated eIF2α protein in the articular chondrocytes regardless of DMM surgery (Figure 7G).

ChIP analysis was performed on AC to determine the interactions between Spi-1 and the ADAMTS-5 promoter. Regardless of whether DMM surgery was performed, the binding of Spi-1 was significantly decreased in the AC of PKR^Col2a1-CreERT^ mice compared to PKR^fl/fl^ mice (Figure 7H).

Immunohistochemistry data showed that the staining of ADAMTS-5 protein was enriched significantly in the articular chondrocytes of DMM mice compared with Sham mice (Figure 7I). PKR conditional knockout from articular chondrocytes decreased the staining of ADAMTS-5 protein in the articular chondrocytes of OA mice (Figure 7I).

## Discussion

Recent research has identified a link between mitochondrial dysfunction-induced mt-dsRNA overexpression and the progression of OA, yet the mechanisms involved remain unclear. In this study, we discovered three significant insights: (1) mitochondrial dysfunction triggered the movement of mt-dsRNAs into the cytosol of chondrocytes. These mt-dsRNAs then activated the PKR signaling pathway to initiate Spi-1-dependent ADAMTS-5 transcription; (2) the increased presence of mt-dsRNAs and the activation of PKR and eIFα in the AC of OA patients and OA mice further corroborate our findings; and (3) PKR conditional knockout from articular chondrocytes alleviates the pathological phenotypes of OA mice. This study sheds light on the mechanisms by which mitochondrial dysfunction contributes to OA development.

Within the scope of OA, there is a recognized link between mitochondrial dysfunction and cellular damage, stemming from disruptions in mitochondrial processes and metabolic shifts. These disruptions include fragmented mitochondrial mass, heightened ROS production, apoptosis, diminished ATP synthesis, and autophagy.^30^^,^^39^^,^^40^^,^^41^^,^^42^ Recently, mtRNAs have garnered attention as strong activators of innate immunity.^29^^,^^35^^,^^43^ The bidirectional transcription of the circular genome leads to the production of long complementary RNAs by mtDNA, which can pair up to create intermolecular dsRNAs.^44^ Upon their release into the cytosol, these mt-dsRNAs are identified by MDA5 and PKR, which then activate the type I IFN response and initiate the processes leading to apoptosis.^24^^,^^29^^,^^35^^,^^43^ Significantly, when conditions such as mitochondrial dysfunction, heightened ROS levels, and DNA damage provoke OA, mt-dsRNAs are released into the cytosol. This release triggers the activation of PKR, which ultimately leads to the demise of chondrocytes.^29^ Our research also identified the release of mt-dsRNAs into the cytosol and the subsequent activation of PKR when chondrocytes were induced to mitochondrial dysfunction. Our research indicates that mt-dsRNAs could be part of the unidentified dsRNAs originating from damaged articular chondrocytes, which play a role in the deterioration of cartilage.

Our research delves into the activation of PKR triggered by mt-dsRNA, offering significant insights into the pathogenesis of OA. Several studies have documented elevated pPKR levels in the AC of OA patients.^45^^,^^46^^,^^47^ Furthermore, PKR activation has been connected to both inflammation and the production of MMP-13 in human articular chondrocytes.^48^ We observed that OA-inducing conditions activate the PKR signaling pathway both *in vitro* and *in vivo*. Our findings indicate that mitochondrial dysfunction facilitates the cytosolic release of mt-dsRNAs, which then engage with PKR to activate the innate immune response. Specifically, PKR has been recognized as the key dsRNA sensor that induces cell death when mitochondrial dysfunction occurs, and it also has a role in the regulation of ISGs. Furthermore, PKR was identified as the principal upstream kinase involved in the phosphorylation of eIF2α during mitochondrial dysfunction.

In an earlier investigation, researchers showed that mitochondrial dysfunction leads to the release of mt-dsRNAs into the extracellular environment, where they are detected by TLR3 at the plasma membrane, triggering changes associated with OA.^29^ Nonetheless, that study did not pinpoint the specific effector through which mt-dsRNA-activated PKR facilitates OA progression. Consequently, our research examined the expression profiles of key proteases involved in the degradation of AC during OA, such as MMP-1, MMP-3, MMP-9, MMP-13, ADAMTS-4, and ADAMTS-5, following oligo A treatment. The findings revealed that oligo A did not alter the mRNA and protein levels of MMP-1, MMP-3, MMP-9, MMP-13, and ADAMTS-4. However, there was a significant up-regulation in the mRNA and protein levels of ADAMTS-5 in response to oligo A treatment. Notably, when we used 2-CM to suppress mt-dsRNA expression or siRNA to reduce PKR expression, the activation of ADAMTS-5 expression due to oligo A treatment was significantly diminished. This suggests that PKR, activated by mt-dsRNAs, is crucial for the up-regulation of ADAMTS-5 expression during OA. To our knowledge, this is the first study to introduce the idea that the efflux of mt-dsRNAs into the cytosol activates PKR, thereby promoting OA development through the activation of ADAMTS-5 expression in chondrocytes, offering deeper insights into the role of mitochondrial dysfunction in OA progression.

The current study has yielded another significant insight by exploring the mechanisms through which PKR activates ADAMTS-5 expression. Our earlier studies have already established the crucial involvement of the transcription factor Spi-1 in triggering ADAMTS-5 expression during OA.^18^^,^^19^ In this study, the alterations of Spi-1 in reaction to mitochondrial dysfunction were examined. oligo A did not affect the mRNA and protein levels of Spi-1. Nonetheless, western blot analysis revealed a notable increase in Spi-1 within the nucleus following oligo A treatment compared to the vehicle control, indicating that Spi-1 protein was accumulated in the nucleus in response to mitochondrial dysfunction, even though the overall Spi-1 expression remained constant with oligo A treatment. Additionally, ChIP data showed that Spi-1 was enriched in the ADAMTS-5 promoter after oligo A treatment. Our findings suggest that PKR might trigger the activation of ADAMTS-5 expression in mouse chondrocytes by facilitating the accumulation of Spi-1 in the nucleus and the enrichment of Spi-1 in the ADAMTS-5 promoter, rather than altering Spi-1 expression levels.

The most important data of current study come from OA mice in response to PKR conditional knockout from articular chondrocytes. PKR is constitutively expressed at low levels in many cell types where it is activated by stress signals, dsRNA, viral infection and pro-inflammatory cytokines.^49^^,^^50^^,^^51^ PKR dysregulation has been implicated in many inflammatory processes and age-related diseases including arthritis,^49^^,^^50^ where PKR activation promotes cartilage breakdown and bone remodeling. Numerous studies reported the increased levels of pPKR in damaged cartilage of OA patients.^45^^,^^46^^,^^47^ Moreover, PKR activation was responsible for inflammation and MMP-13 secretion in human articular chondrocytes.^48^ We also found the activation of the PKR signaling pathway by OA-eliciting conditions both *in vitro* and *in vivo*. However, no studies have investigated the change of OA phenotype in mice with PKR deletion from cartilage so far. In the current study, using Cre-loxP system and conditional knockout mice model, we deleted PKR from AC of mice which then undergo DMM surgery. The robust data showed the significant alleviation of OA phenotype, including cartilage destruction, subchondral bone sclerosis, and ADAMTS-5 expression activation. To our knowledge, the current study is first to make attempts to delete PKR completely from AC of mice via Cre-loxP system and to provide direct evidences that PKR plays an important role in OA phenotype, highlighting the potential of PKR developed as a promising target of OA drugs.

**Figure 8:**
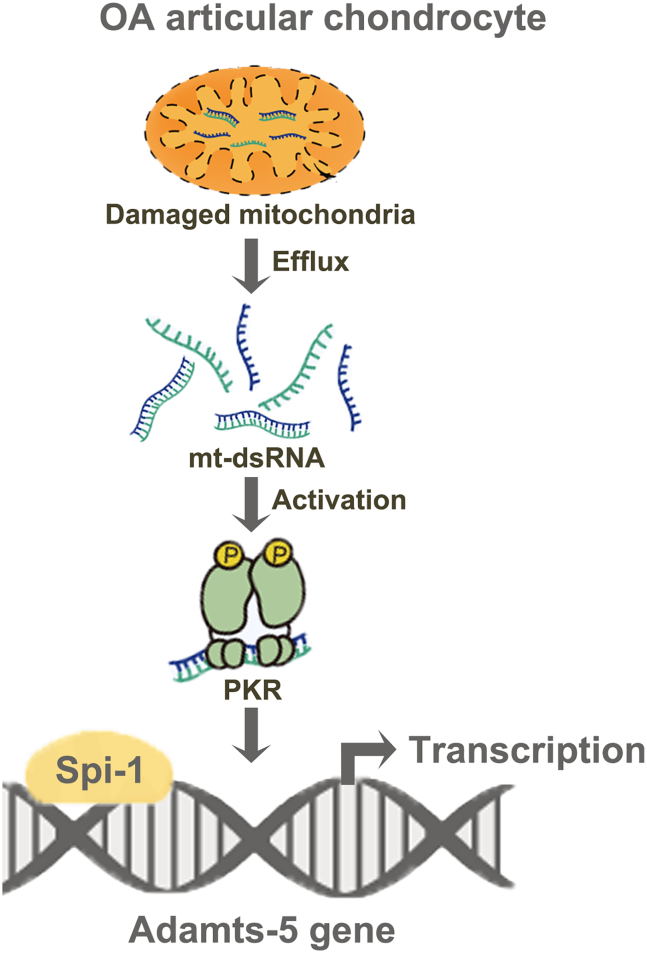
Diagram for the mechanisms underlying ADAMTS-5 expression activated by mt-dsRNA-PKR-Spi-1 axis in OA chondrocytes Mitochondrial dysfunction leads to the release of mt-dsRNAs into the cytosol of chondrocytes. These mt-dsRNAs then activate Spi-1-dependent ADAMTS-5 expression via the PKR signaling pathway. Consequently, ADAMTS-5 degrades the AC, resulting in the manifestation of the OA phenotype.

In summary, the current research indicates that mitochondrial dysfuntion causes mt-dsRNAs to be released into the cytosol of chondrocytes. These mt-dsRNAs then activated the PKR signaling pathway to initiate Spi-1-dependent ADAMTS-5 transcription. Consequently, ADAMTS-5 contributes to the degradation of AC, resulting in the manifestation of the OA phenotype (Figure 8). Our investigation sheds light on the role of mitochondrial dysfunction in chondrocytes in the progression of OA.

### Limitations of the study

Our study has certain limitations. We did not compare the expression levels of mt-dsRNAs between healthy individuals and OA patients because normal cartilage sampling may be unethical or is often impractical in subjects with normal joints. Instead, we used OA patients in stage 1 as baseline. The data from OA patients in stage 2 and 3 were compared with that from OA patients in stage 1. We also utilized a mouse model to replicate OA caused by surgery. Unlike this, human OA usually emerges slowly over several years due to routine activities, without any distinct traumatic incident. Despite this, the OA model using mice that was utilized in this research is broadly accepted in the field of OA studies^52^^,^^53^ due to its simplicity in examining the entire disease process. According to the previous research,^54^ there is a noted rise in PKR levels as age progresses. Investigating spontaneous OA through a large animal model could provide valuable insights into the role of the mtRNA-PKR regulatory axis.

## Resource availability

### Lead contact

Further information and requests for resources and reagents should be directed to and will be fulfilled by the lead contact, Deqiang Li (deqiang_lee@163.com).

### Materials availability

This study did not generate new unique reagents.

### Data and code availability

• The data that support the findings of this study are openly available in Mendeley at https://doi.org/10.17632/99w5n6v67z.1.
• This study does not report original code.
• Any additional information required to reanalyze the data reported in this study is available from the lead contact upon request.

## Acknowledgments

This study was supported by the Project of 10.13039/501100001809National Natural Science Foundation of China (82372398).

## Author contributions

D.L. conceived and designed the research. Y.M., S.Y., Y.W., B.L., R.T., H.Y., L.M., and Y.Z. performed the experiments and collected the data. L.M., Y.Z., and D.L. analyzed the data. D.L. wrote the manuscript.

## Declaration of interests

The authors declare no competing interests.

## STAR★Methods

### Key resources table

**Table:** 

REAGENT or RESOURCE	SOURCE	IDENTIFIER
**Antibodies**		

Anti-pPKR	Novus	NB100-82156; RRID: AB_1144605
Anti-PKR	Novus	N00005610-M02; RRID: AB_828845
Anti-peIF2α	Novus	NB100-81896; RRID: AB_1144477
Anti-eIF2α	Novus	NBP3-14927; RRID: AB_3598073
Anti-ADAMTS-5	Thermo Fisher Scientific	PA5-27165; RRID: AB_2544641
Anti-Flag-tag	Cell Signaling Technology	#14793; RRID: AB_2572291
Anti-Spi-1	Cell Signaling Technology	#2258; RRID: AB_2186909
Anti-β-tubulin	Cell Signaling Technology	#2146; RRID: AB_2210545
Anti-Spi-1	Thermo Fisher Scientific	MA5-15064; RRID: AB_10986949
Anti-pPKR	Thermo Fisher Scientific	44-668G; RRID: AB_2533716
Isotype IgG	Cell Signaling Technology	#61656; RRID: AB_2799613
secondary antibody	Cell Signaling Technology	#7074; RRID: AB_2099233

**Chemicals, peptides, and recombinant proteins**		

oligomycin A	Sigma-Aldrich	75351
2-C-methyladenosine	MedChemExpress	HY-125371
Recombinant human Spi-1 protein	CUSABIO	CSB-EP022567HU
Recombinant PKR protein	CUSABIO	CSB-EP007511HU
tamoxifen	Sigma-Aldrich	T5648
protease	Sigma-Aldrich	D4693
collagenase	Gibco	17100-017
DCFH-DA	Sigma-Aldrich	D6883
TRIzol LS	Invitrogen	10296-010
RIPA buffer	Pierce	89900
protease inhibitors cocktail	Pierce	78438
Western Lightning Plus-ECL	Perkin Elmer	NEL105001EAa
PrimeSTAR Max DNA Polymerase	Takara	R045A
Proteinase K	Roche	03115879001
DMEM	Gibco	C11995500BT

**Critical commercial assays**		

MitoProbe JC-1 Assay Kit	Invitrogen	M34152
protein fractionation kit	Pierce	78840
RevertAid Premium First Strand cDNA Synthesis Kit	Thermo Fisher Scientific	K1651
FastStart Universal SYBR Green Master	Roche	4913850001
Lenti-Pac HIV qRT-PCR Titration Kit	Genecopia	LT006
horseradish peroxidase-streptavidin detection system	Abcam	ab93677
Lenti-Pac Lentivirus Concentration Solution	Genecopia	LT007

**Deposited data**		

Original data	Mendeley	https://doi.org/10.17632/99w5n6v67z.1

**Experimental models: Cell lines**		

SW1353	ATCC	HTB-94
CHON-001	ATCC	CRL-2846
HEK293T	ATCC	CRL-11268

**Experimental models: Organisms/strains**		

C57BL/6 mice	SIPPR-BK Laboratory Animal Co. Ltd	N/A
Transgenic Col2a1-CreERT mice	Jackson Laboratory	006774

**Software and algorithms**		

GraphPad Prism	GraphPad	https://www.graphpad.com/scientificsoftware/prism/
Image J	NIH	https://imagej.nih.gov/ij/

### Experimental model and study participant details

#### Animals

All mice were kept in the animal facility at Shandong University’s Qilu Hospital, maintained under SPF conditions. The protocol followed for all experiments was approved by the Qilu Hospital Animal Care and Use Committee of Shandong University (DWLL-2023-165). All animal experiments were performed in compliance with the National Research Council’s Guide for the Care and Use of Laboratory Animals and the ARRIVE guidelines. PKR^flox/flox^ mice were generated by inserting loxP sequences into upstream of exon 5 and downstream of exon 7 of PKR gene. PKR^Col2a1-CreERT^ mice were produced by crossing PKR^flox/flox^ mice with transgenic Col2a1-CreERT mice. To delete PKR from articular chondrocytes, tamoxifen was administered intraperitoneally (1 mg/10 g body weight) to PKR^Col2a1-CreERT^ mice. The treatment lasted for five consecutive days.

#### Destabilisation of medial meniscus (DMM)-induced OA mice model

Each group consisted of six animals. The DMM surgery was conducted following the procedures detailed in our previous publication.^18^^,^^19^ Male C57BL/6 mice, aged 8 weeks, were anesthetized using isoflurane. During the surgery on the right knee joint, a cut was made on the medial meniscotibial ligament, which connects the medial meniscus to the tibial plateau. The sham operation followed the same procedure, except the ligament was not cut. After the DMM surgery, the mice regained full mobility. The mice were euthanized after the experiments, and their knee joints were collected eight weeks post-DMM surgery.

#### Cell cultures

Between January 2021 and December 2022, cartilage samples were collected from 14 OA patients (10 women and 4 men, with an average age of 71.7±4.8 years) undergoing total knee replacement (TKR) surgery at the Department of Orthopedics (Qilu Hospital of Shandong University, Jinan, China) according to the principles expressed in the Declaration of Helsinki. The experimental procedures were approved by the Ethics Committee of Qilu Hospital of Shandong University (approval number KYLL-202007-072). All patients had signed informed consent for sample collection and data analysis prior to surgery. To isolate primary chondrocyte, the cartilage was finely chopped and digested in DMEM supplemented with protease for one hour at 37°C in a 5% CO_2_ environment. Afterward, the cartilage was rinsed with PBS and subjected to a 0.3% (w/v) collagenase treatment for three hours to break down the collagen. The cells were then filtered through a 70-μm cell strainer. To maintain phenotypic stability, cells from the second and third passages were used.

Human cell lines SW1353 (RRID: CVCL_0543) and CHON-001 (RRID: CVCL_C462) were cultured in DMEM with an addition of 10% (v/v) FBS. These cell lines were maintained at 37°C in a 5% CO_2_ environment, with passaging occurring at a 1:5 ratio every 2 to 3 days. SW1353 and CHON-001 cell lines were verified via Short Tandem Repeat (STR) at the beginning (99.78%, and 99.64%) and end (99.75%, and 99.88%) of the study. These two cell lines were confirmed to be free of mycoplasma contamination via PCR.

#### *In vitro* cartilage culture of OA patients

The cartilage from OA patients was initially sliced in PBS supplemented with Antibiotic-Antimycotic (1%). The cartilage was rinsed with PBS, placed into a 48-well plate, and cultured in DMEM supplemented with FBS (10%) and Antibiotic-Antimycotic (1%) for further use.

### Method details

#### Reagents and treatments

To trigger mitochondrial dysfunction, chondrocytes were exposed to 30 μg/ml of oligomycin A (Oligo A) for a duration of 24 hours. Additionally, cells were treated with 50 μM of 2-C-methyladenosine (2-CM) for 48 hours to suppress mtRNA expression. Recombinant human Spi-1 protein and PKR protein were added to the cell culture to recover the function of Spi-1 and PKR blunted by siRNA.

#### Mitochondrial membrane potential

The MitoProbe JC-1 Assay Kit was utilized to assess the mitochondrial membrane potential. Initially, cells were rinsed once with PBS and then incubated in a pre-heated medium containing JC-1 dye for 30 minutes at 37°C in an incubator with 5% CO_2_. Following this, the cells underwent three PBS washes, and JC-1 staining was detected using flow cytometry.

#### Intracellular ROS production

The cells underwent a single wash with PBS after being treated with Oligo A. They were then incubated in medium supplemented with DCFH-DA for 30 minutes. Following this, PBS was used to wash the cells three times, and the fluorescence from DCFH-DA (excitation 495 nm/emission 529 nm) was observed via an inverted fluorescence microscope (Nikon, Ti2-U).

#### Extraction of cytoplasmic RNA

A protein fractionation kit was utilized to extract cytoplasmic RNA according to the instructions provided by the manufacturer. The cytosolic fraction was then treated with TRIzol LS in a 3:1 ratio to facilitate RNA extraction.

#### RT-qPCR

Cells and articular cartilage were used to extract RNA with the help of Trizol Reagent. The RevertAid Premium First Strand cDNA Synthesis Kit was employed for reverse transcription of 500 ng of RNA. PCR was conducted using an Applied Biosystems 7500HT real-time PCR machine, and the FastStart Universal SYBR Green Master was utilized. Table S1 contains the primer sequences. Gene expression was evaluated using the 2^-ΔΔCt^ method, with normalization to β-tubulin, which acted as a housekeeping control. The data presented are fold changes compared to the control group.

#### Western blot

The total protein was extracted using RIPA buffer enhanced with a protease inhibitors cocktail. A 25-μg sample of the isolated proteins was separated using SDS-PAGE and subsequently transferred onto 0.22-μm PVDF membranes (Millipore, ISEQ00010) through the process of electroblotting. The membranes were then blocked with 5% BSA-PBS for an hour before being exposed to primary antibodies. Following a wash with TBS-0.1% Tween 20 (TBST), the membranes were placed in a dark environment for 1 hour with a secondary antibody. The protein bands were visualized using Perkin Elmer Western Lightning Plus-ECL, and the protein quantities were measured with Image J software.

#### PKR mutagenesis

PKR plasmids containing point mutations at critical phosphorylation sites were created using PCR. The primer sequences for T446A were 5′-AATGATGGAAAGCGAGCAAGGAGTAAGGGAA-3′ (F) and 5′-TTCCCTTACTCCTTGCTCGCTTTCCATCATT-3′ (R). For T451A, the primer sequences were 5′-ACAAGGAGTAAGGGAGCTTTGCGATACATGA-3′ (F) and 5′-TCATGTATCGCAAAGCTCCCTTACTCCTTGT-3′ (R). To generate plasmids with a point mutation, PrimeSTAR Max DNA Polymerase was employed. The PCR conditions adhered to the manufacturer’s guidelines. Sanger sequencing was used to verify the point mutations.

#### PKR formaldehyde crosslinking immunoprecipitation (fCLIP)

Cells were treated with 0.1% paraformaldehyde at room temperature for 10 minutes to fix them. Glycine was then added to quench the reaction, and the cells were incubated for another 10 minutes at room temperature. The crosslinked cells were lysed using the fCLIP lysis buffer of 20 mM Tris-HCl (pH 7.5) supplemented with 15 mM NaCl, 10 mM EDTA, 0.5% NP-40, 0.1% Triton X-100, and 0.1% Sodium deoxycholate, followed by sonication three times for 5 minutes each. The lysate underwent immunoprecipitation with a PKR antibody for 3 hours at 4°C. To elute PKR-RNA complexes from the beads, a buffer of 200 mM Tris-HCl (pH 7.4) supplemented with 100 mM NaCl, 20 mM EDTA, 2% SDS, and 7 M Urea was used. The RNA was then separated from PKR by incubating at 65°C for at least 12 hours with 20 mg/ml Proteinase K to de-crosslink it.

#### Chromatin immunoprecipitation (ChIP)

To fix the cells, they were exposed to 1% formaldehyde in the growth medium for a duration of 10 minutes at room temperature. After washing with ice-cold PBS, the cells were lysed in a solution containing 1% SDS and a protease inhibitor cocktail. The lysates were then sonicated to obtain chromatin fragments. The chromatin solution was precleared for an hour at 4°C with gentle stirring using protein G agarose beads. The remaining chromatin was immunoprecipitated overnight at 4°C with head-over-tail rotation using 5 μg of anti-Spi-1. Isotype IgG served as a negative control. Chromatin/antibody complexes were collected using protein G-conjugated magnetic beads and then eluted with 50 μl of elution buffer (0.1 M NaHCO3 and 1% SDS). Proteinase K and RNase A were used to treat the immunoprecipitated chromatin and input samples, respectively, before purification through DNA isolation columns. Subsequently, the samples were analyzed by qPCR. The primer sequences for the Spi-1 binding site on the mouse Adamts-5 promoter were 5′-CCTTCAGGAGATGAGGTAAG-3′ (F) and 5′-TGCCATCCTGCAATGGCAAT-3′ (R). For the human ADAMTS-5 promoter, the primer sequences were 5′-ATGTCACCCGCGTACTAATA-3′ (F) and 5′-AATCTGTCCCTTACTCCCAG-3′ (R).

#### Lentivirus vector

Genecopia offers expression plasmid vectors that include the coding sequence (CDS) for human PKR (EX-A0265-M35) and shRNAs targeting human PKR (HSH106978-LVRH1GP), Spi-1 (HSH017663-LVRH1GP), and ADAMTS-5 (HSH169578-LVRH1GP). These were sub-cloned into pReceiver-Lv102 (Genecopia) to create the lentivirus expression plasmid. To produce recombinant lentiviruses, human embryonic kidney 293T cells were co-transfected with the lentivirus expression and packaging plasmids (Genecopia, LT002). The supernatant containing the lentivirus particles was concentrated using Genecopia’s Lenti-Pac Lentivirus Concentration Solution. The Lenti-Pac HIV qRT-PCR Titration Kit was utilized to assess the titers of viral particles, which were subsequently portioned into aliquots and kept at -80°C until required.

#### Histopathological evaluation

Knee specimens from mice were decalcified for four weeks in a 12.5% PBS-ethylene diamine tetraacetic acid (EDTA) solution subsequent to their fixation in 10% formaldehyde for a duration of 48 hours. Following a 5 μm sagittal sectioning, rinsing with tap water, and implantation in cassettes, the joints underwent dehydration and clearing processes. Stained sections were examined for matrix proteoglycan and overall articular cartilage morphology using Safranin O/Fast Green. The OARSI grading system^55^ was used to grade articular cartilage under a microscope. In the proximal tibia, total cartilage area and uncalcified cartilage area were evaluated in accordance with the criteria outlined in the prior publication.^56^ In order to determine the subchondral bone plate (SBP) thickness, five uniformly distributed thicknesses were measured on the medial tibial plateau.^57^ Two experienced investigators reviewed all slides in a blind manner.

#### Immunohistochemistry

The sections embedded in paraffin were first de-paraffinized using xylene and then dehydrated with ethanol. To inhibit endogenous peroxidase activity, 0.3% hydrogen peroxide was applied. Antigen retrieval was performed by treating the sections with proteinase K in PBS for 20 minutes. The sections were then blocked with PBS containing 4% horse serum for 30 minutes. Subsequently, the sections were kept at 4°C overnight to allow incubation with primary antibodies. Subsequently, the sections were treated with biotinylated IgG for 90 minutes. For the immunohistochemistry staining, a horseradish peroxidase-streptavidin detection system was used to visualize the immuno-activity. Isotype IgG served as the negative control.

### Quantification and statistical analysis

Data (mean ± std) was representative of three independent experiments. To compare two groups, an unpaired Student’s t-test was conducted. For analyses involving multiple groups, one-way and two-way ANOVA were employed. To determine significant differences in these ANOVAs, Tukey and Sidak tests were applied, respectively. Spearman’s method was used for correlation analysis. Utilizing GraphPad Prism 8 software, graphs were constructed and statistical analyses were executed. A *p* < 0.05 was regarded as an indicator of statistical significance. All of the statistical details of experiments can be found in the figure legends.

## Supplemental information

Method S1. Supplemental methods

Document S1. Figures S1–S9 and Tables S1 and S2

Document S2. Gels and Blots
