# Supplementary material for: Mitochondrial dysfunction activates ADAMTS-5 expression via mt-dsRNA-PKR-Spi-1 axis in osteoarthritic chondrocytes
Source: iScience. 2026 May 20;29(6):115980. doi: 10.1016/j.isci.2026.115980 (PMC13214264; doi:10.1016/j.isci.2026.115980)
Supplement: Method S1. Supplemental methods [file mmc1.pdf]

iScience, Volume *29*

Supplemental information

Mitochondrial dysfunction activates ADAMTS-5 expression via mt-dsRNA-PKR-Spi-1 axis in osteoarthritic chondrocytes

Yulong Mu, Shuaichen Yan, Yizhe Wang, Boning Liang, Rui Tang, Huapu Yang, Liang Ma, Yuankai Zhang, and Deqiang Li

Supplementary materials and methods

Reagents and treatments

Recombinant human Spi-1 protein (CUSABIO, CSB-EP022567HU) and PKR protein (CUSABIO, CSB-EP007511HU) were added to the cell culture medium to recover the function of Spi-1 and PKR blunted by siRNA. Z-VAD-FMK (50 μM) (R&D systems, FMK001) was employed as pan-caspase inhibitor.

Anterior cruciate ligament transection (ACLT)-induced OA mice model

All mice were housed in the animal facility of Shandong University, Qilu Hospital, under SPF conditions. The Qilu Hospital Animal Care and Use Committee of Shandong University authorized the protocol that all experiments adhered to. All animal experiments are conducted in accordance with the National Research Council's Guide for the Care and Use of Laboratory Animals and the ARRIVE criteria. Male C57BL/6 mice were acquired from SIPPR-BK Laboratory Animal Co. Ltd in Shanghai, China. Mice weighing between 20 and 22g were maintained at temperatures between 20 and 24°C with a 12h light–dark cycle and relative humidity ranging from 50% to 60%. Water and food were available throughout the entire duration of the experiment.

According to the standard protocols elaborated previously [1], OA mouse models were generated with an ACLT operation to mimic mechanical instability. Specifically, after intraperitoneal anesthesia with 5% chloral hydrate, mice underwent a longitudinal cutaneous incision in the right knee. The ACL was transected under a surgical microscope with an ophthalmic scalpel. The sham operation followed the identical protocol, with the exception that ligament sectioning was omitted. Mice were restored to complete locomotion after ACLT surgery. The mice were euthanized subsequent to the experiments, and the knee joints were harvested at week 8 after undergoing ACLT surgery.

References

1. Glasson, S.S., Blanchet, T.J., and Morris, E.A. (2007). The surgical destabilization of the medial meniscus (DMM) model of osteoarthritis in the 129/SvEv mouse. Osteoarthritis Cartilage *15*, 1061-1069. 10.1016/j.joca.2007.03.006.
