# Supplementary material for: Mitochondrial dysfunction activates ADAMTS-5 expression via mt-dsRNA-PKR-Spi-1 axis in osteoarthritic chondrocytes
Source: iScience. 2026 May 20;29(6):115980. doi: 10.1016/j.isci.2026.115980 (PMC13214264; doi:10.1016/j.isci.2026.115980)
Supplement: Document S1. Figures S1–S9 and Tables S1 and S2 [file mmc2.pdf]

Supplementary figures and legends

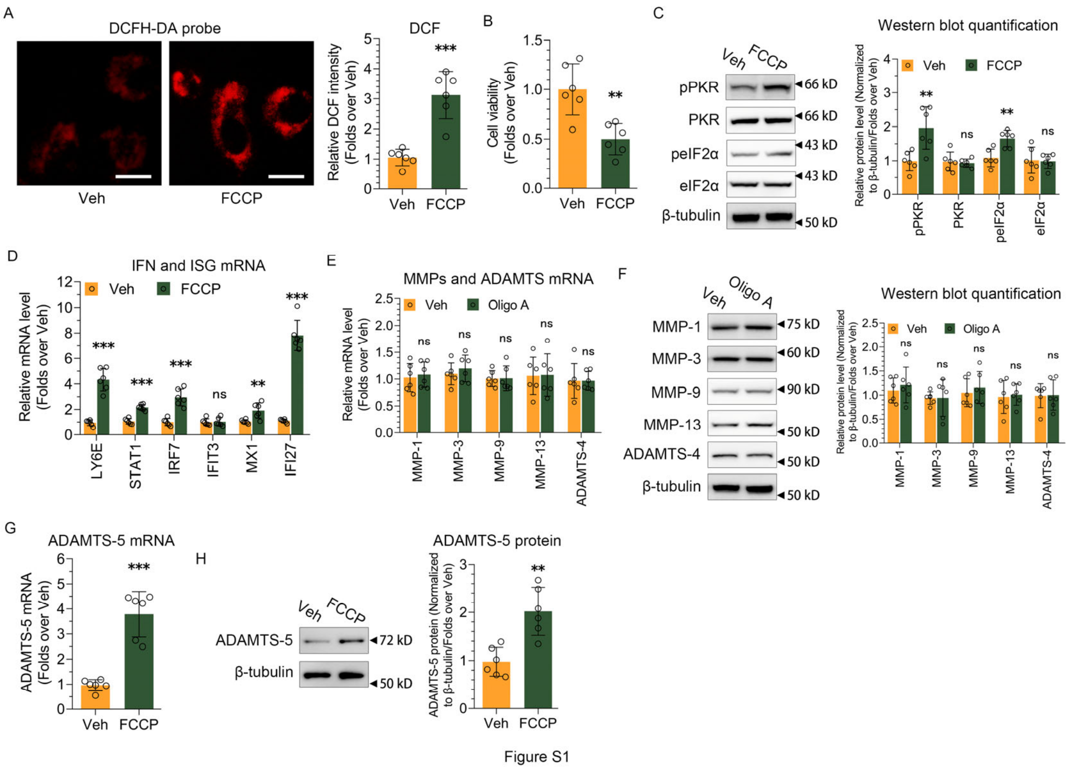

Figure S1: Mitochondrial stressor induces PKR activation and up-regulation of ADAMTS-5 expression in chondrocytes

(A-C) The effect of mitochondrial respiratory chain inhibition by FCCP (1 μM) on ROS production (A, n=6), cell viability (B, n=6), and PKR signaling (C) in SW1353 cell line. Scale bar in (A), 20 μm. Quantification is shown on the right. (D) ISGs mRNA expressions in response to FCCP treatment in SW1353 cell line (n=6). (E and F) RT-qPCR and immunoblotting analysis of mRNA (E, n=6) and protein (F) expression of MMP-1, MMP-3, MMP-9, MMP-13 and ADAMTS-4 in response to Oligo A treatment in SW1353 cell line. Quantification of immunoblotting is shown on the right (n=6). (G and H) RT-qPCR and immunoblotting analysis of ADAMTS-5 mRNA (G, n=6) and protein (H) expression in response to FCCP treatment in SW1353 cell line. Quantification of immunoblotting is shown on the right (n=6). Data (mean ± std) was representative of three independent experiments. An unpaired Student's t-test was conducted to compare two groups. *: $p<0.05$, **: $p<0.01$, ***: $p<0.001$, ns: no significance.

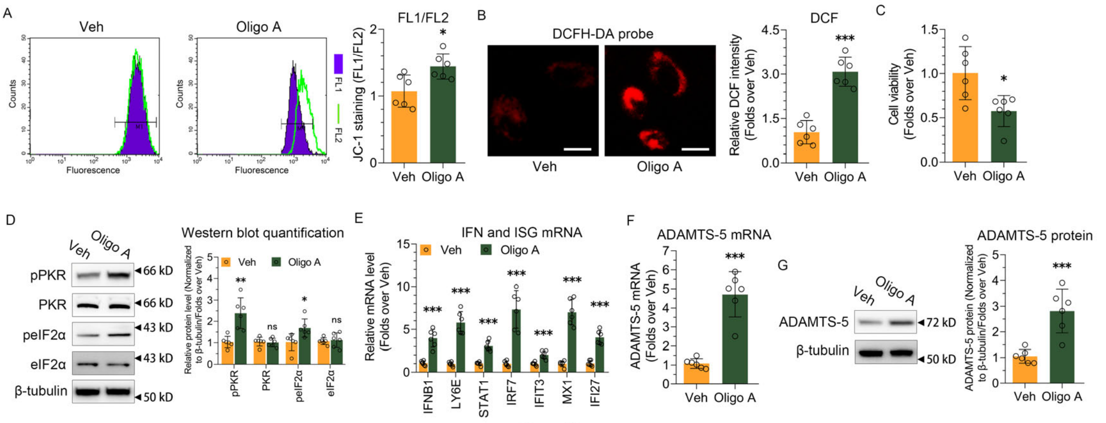

Figure S2

Figure S2: Mitochondrial dysfunction activates ADAMTS-5 expression in human chondrocyte cell line CHON-001

(A-D) The effect of mitochondrial respiratory chain inhibition by Oligo A (30 mg/mL) on the mitochondrial membrane potential (A, n=6), ROS production (B, n=6), cell viability (C, n=6), and PKR signaling (D) in CHON-001 cell line. Scale bar in (B), 20 μm. Quantification is shown on the right (n=6). (E) ISGs mRNA expressions in response to Oligo A treatment in CHON-001 cell line. (F and G) RT-qPCR and immunoblotting analysis of ADAMTS-5 mRNA (F, n=6) and protein (G) expression in response to Oligo A treatment in CHON-001 cell line. Quantification of immunoblotting is shown on the right (n=6). Data (mean ± std) was representative of three independent experiments. An unpaired Student's t-test was conducted to compare two groups. *: p<0.05, **: p<0.01, ***: p<0.001, ns: no significance.

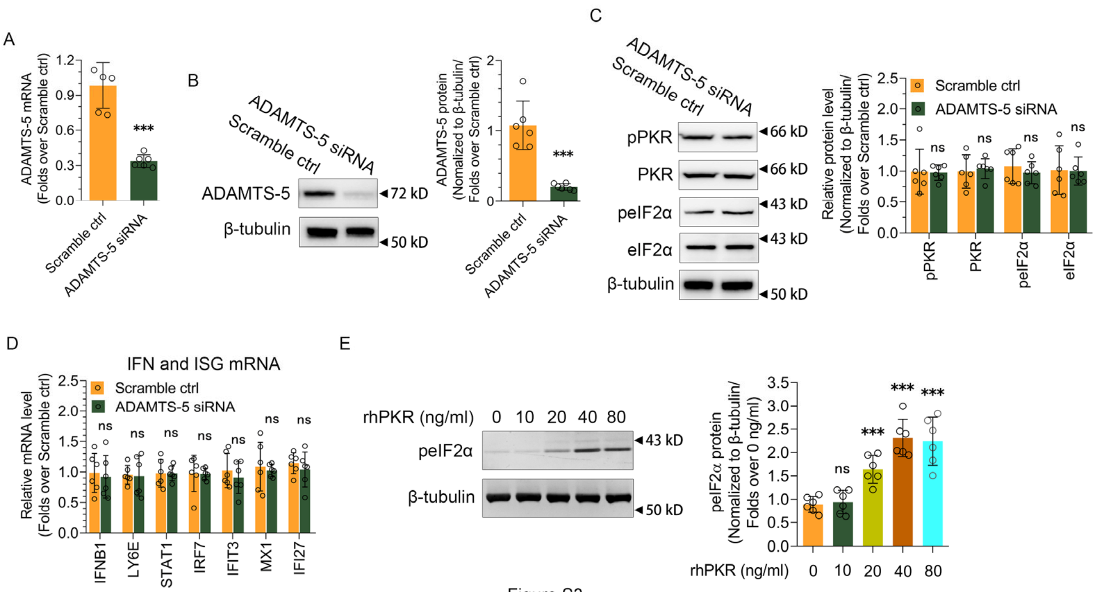

Figure S3

Figure S3: PKR signaling pathway remains unchanged in response to ADAMTS-5 knockdown by siRNA

(A and B) RT-qPCR and immunoblotting analysis of ADAMTS-5 mRNA (A, n=6) and protein (B) expression in response to siRNA-mediated ADAMTS-5 knockdown in SW1353 cell line. Quantification of immunoblotting is shown on the right (n=6). (C) Immunoblotting analysis of PKR and eIF2α phosphorylation in response to ADAMTS-5 knockdown in SW1353 cell line. Quantification is shown on the right (n=6). (D) IFNB1 and ISG mRNA expressions in response to ADAMTS-5 knockdown in SW1353 cell line (n=6). (E) Immunoblotting analysis of phosphorylated eIF2α protein (A, n=6) expression in response to recombinant human PKR protein treatment in dose-dependent manner in SW1353 cell line. Data (mean ± std) was representative of three independent experiments. To compare two groups, an unpaired Student's t-test was conducted in (A, B, C, and D). For analyses involving multiple groups, one-way ANOVA were employed (E). *: $p<0.05$, **: $p<0.01$, ***: $p<0.001$, ns: no significance.

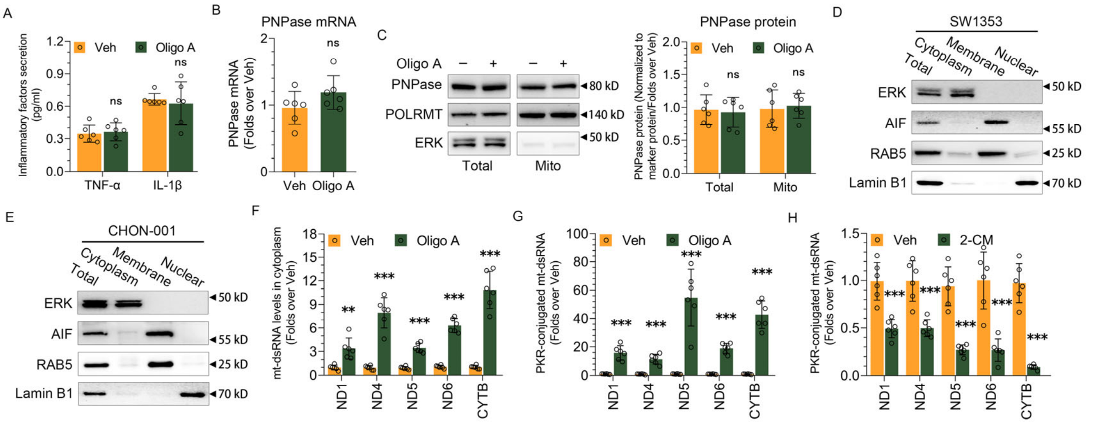

Figure S4

Figure S4: Mitochondrial dysfunction induces the cytosolic efflux of mt-dsRNAs to activate PKR in CHON-001 cell line

(A) TNFα and IL-1β levels of in the medium of SW1353 cell line in response to Oligo A treatment examined by ELISA (n=6). (B and C) RT-qPCR and immunoblotting analysis of PNPase mRNA (B, n=6) and protein (C) expression in response to Oligo A treatment in SW1353 cell line. Quantification of immunoblotting is shown on the right (n=6). (D and E) Immunoblotting analysis of protein expressions of ERK, AIF, RAB5, and Lamin B1 in the cytoplasm, membrane, and nuclear of SW1353 (D) and CHON-001 (E) cell lines. (F) mtRNA levels in the cytoplasm of CHON-001 cell line in response to Oligo A treatment examined by RT-qPCR (n=6). (G) PKR-mtRNA interaction upon Oligo A treatment in CHON-001 cell line examined by PKR fCLIP-qPCR analysis (n=6). (H) PKR-mtRNA interaction upon 2-CM treatment in CHON-001 cell line examined by PKR fCLIP-qPCR analysis (n=6). Data (mean ± std) was representative of three independent experiments. An unpaired Student's t-test was conducted to compare two groups. *: $p<0.05$, **: $p<0.01$, ***: $p<0.001$, ns: no significance.

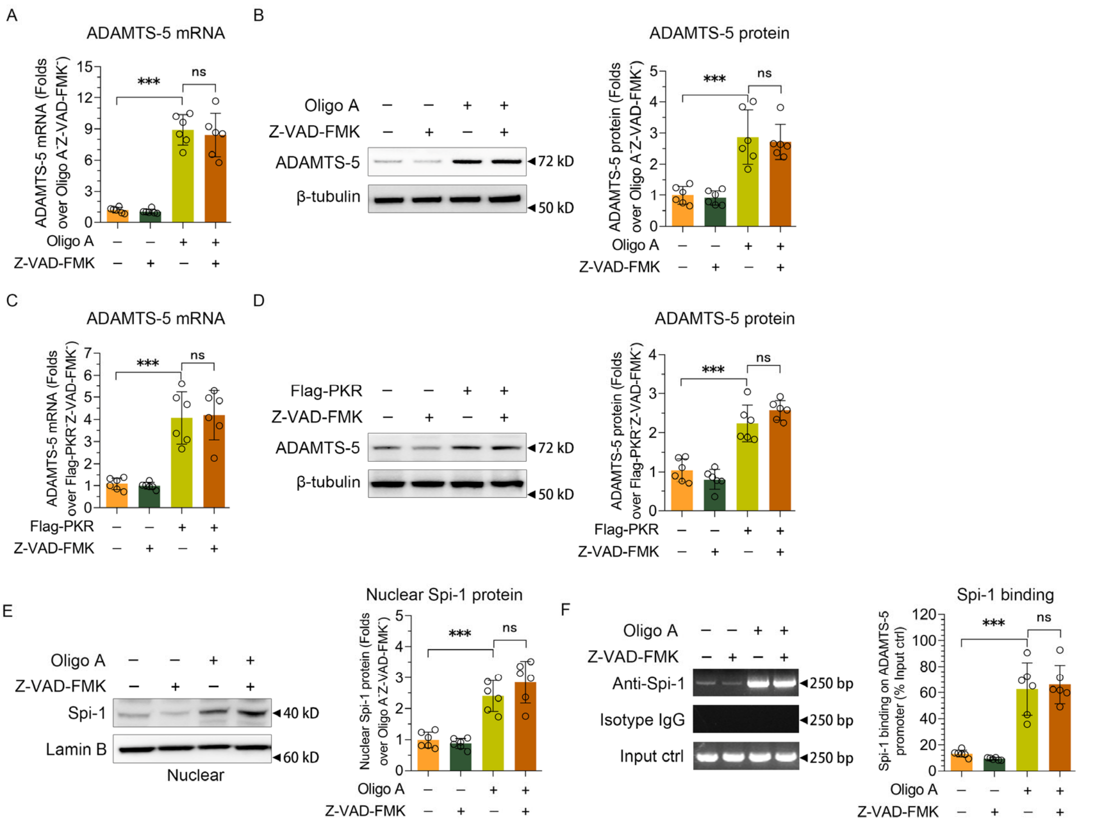

Figure S5

Figure S5: Caspase-mediated cell death is not involved in the activation of ADAMTS-5 expression, the accumulation of Spi-1 in the nuclear and the enrichment of Spi-1 on ADAMTS-5 gene promoter induced by mitochondrial dysfunction

(A and B) RT-qPCR and immunoblotting analysis of ADAMTS-5 mRNA (A, n=6) and protein (B) expression in response to Oligo A and/or pan-caspase inhibitor Z-VAD-FMK in SW1353 cell line. Quantification of immunoblotting is shown on the right (n=6). (C and D) RT-qPCR and immunoblotting analysis of ADAMTS-5 mRNA (C, n=6) and protein (D) expression in response to PKR overexpression and/or pan-caspase inhibitor Z-VAD-FMK in SW1353 cell line. (E) Immunoblotting analysis of ADAMTS-5 protein abundance in the nuclear in response to Oligo A and/or pan-caspase inhibitor Z-VAD-FMK in SW1353 cell line. Quantification of immunoblotting is shown on the right (n=6). (F) The binding of Spi-1 on the ADAMTS-5 promoter in response to Oligo A and/or pan-caspase inhibitor Z-VAD-FMK was evaluated using ChIP, with quantification on the right (n=6). Data (mean ± std) was representative of three independent experiments. For analyses involving multiple groups, two-way ANOVA were employed. *: $p<0.05$, **: $p<0.01$, ***: $p<0.001$, ns: no significance.

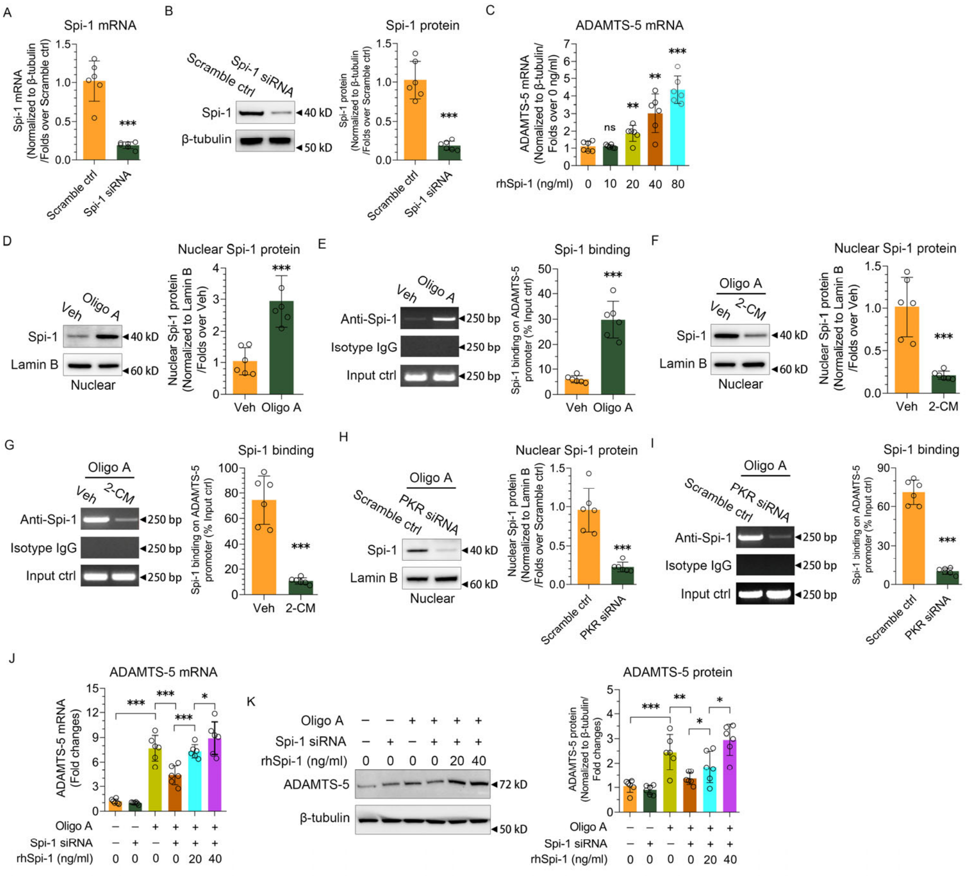

Figure S6

Figure S6: Mitochondrial dysfunction-induced cytosolic efflux of mt-dsRNAs enhances Spi-1 binding on ADAMTS-5 promoter to activate ADAMTS-5 expression in CHON-001 cell line.

(A and B) RT-qPCR and immunoblotting of Spi-1 mRNA (A, n=6) and protein (B) expression in response to Oligo A. Quantification of immunoblotting is shown on the right (n=6). (C) RT-qPCR of ADAMTS-5 mRNA (A, n=6) expression in response to recombinant human Spi-1 protein in dose-dependent manner. (D) Immunoblotting of Spi-1 protein expression in the nuclear of CHON-001 cell line in response to Oligo A. Quantification of immunoblotting is shown on the right (n=6). (E) Spi-1 binding on ADAMTS-5 promoter in response to Oligo A examined by ChIP. Quantification is shown on the right (n=6). (F) Immunoblotting of Spi-1 protein expression in the nuclear of CHON-001 cell line in response to Oligo A and 2-CM. Quantification of immunoblotting is shown on the right (n=6). (G) Spi-1 binding on ADAMTS-5 promoter in response to Oligo A and 2-CM examined by ChIP. Quantification is shown on the right (n=6). (H) Immunoblotting of Spi-1 protein expression in the nuclear of CHON-001 cell line in response to Oligo A treatment and PKR knockdown. Quantification of immunoblotting is shown on the right (n=6). (I) Spi-1 binding on ADAMTS-5 promoter in response to Oligo A treatment and PKR knockdown examined by ChIP. Quantification is shown on the right (n=6). (J and K) RT-qPCR and immunoblotting of ADAMTS-5 mRNA (J, n=6) and protein (K) expression in response to Oligo A treatment, Spi-1 knockdown and/or Spi-1 protein treatment. Quantification of immunoblotting is shown on the right (n=6). Data (mean ± std) was representative of three independent experiments. To compare two groups, an unpaired Student's t-test was conducted in (A, B, D, E, F, G, H, and I). For analyses involving multiple groups, one-way (C) and two-way (J and K) ANOVA were employed. *: p<0.05, **: p<0.01, ***: p<0.001, ns: no significance.

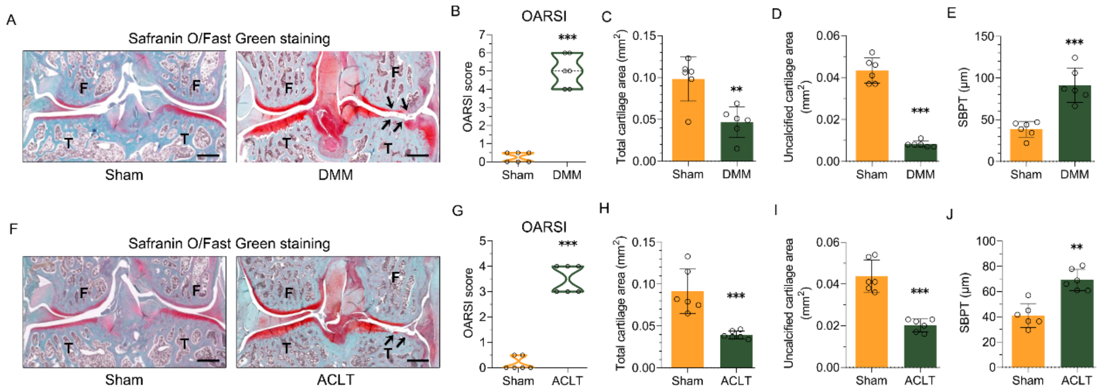

Figure S7

Figure S7: Destabilisation of medial meniscus (DMM)- and Anterior cruciate ligament transection (ACLT)-induced OA mice models.

(A) Representative photomicrographs of knee joint sections obtained from mice undergoing Sham or DMM surgery, stained with Safranin O/fast green. The arrows represent cartilage damage. The bar of scale is 100 μm. (B) OARSI score quantification of the severity of OA in mice, as shown in (A); n=6 per group. (C) The total cartilage area as quantified in (A); n=6 per group. (D) Uncalcified cartilage area quantification as shown in (A); n=6 per group. (E) Subchondral bone plat thickness (SBPT) quantification for mice depicted in (A); n=6 per group. (F) Representative photomicrographs of knee joint sections obtained from mice undergoing Sham or ACLT surgery, stained with Safranin O/fast green. The arrows represent cartilage damage. The bar of scale is 100 μm. (G) OARSI score quantification of the severity of OA in mice, as shown in (F); n=6 per group. (H) The total cartilage area as quantified in (F); n=6 per group. (I) Uncalcified cartilage area quantification as shown in (F); n=6 per group. (J) Subchondral bone plat thickness (SBPT) quantification for mice depicted in (F); n=6 per group. Data (mean ± std) was representative of three independent experiments. An unpaired Student's t-test was conducted to compare two groups. *: $p<0.05$, **: $p<0.01$, ***: $p<0.001$, ns: no significance.

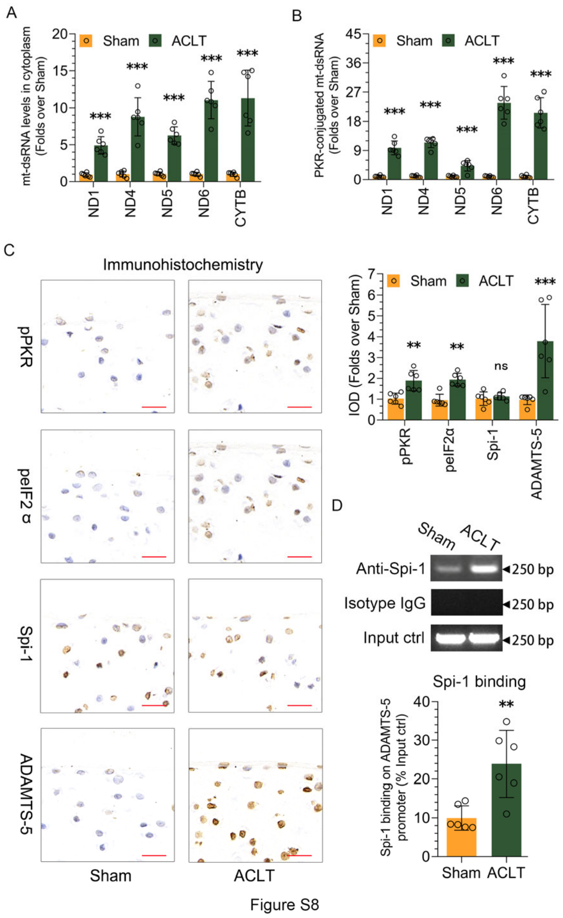

Figure S8: mt-dsRNAs expression up-regulation and PKR activation in damaged cartilage of OA mice

(A) mtRNA levels in the cytoplasm of chondrocytes from articular cartilage of Sham and ACLT mice examined by RT-qPCR (n=6). (B) PKR-mtRNA interaction in the chondrocytes from articular cartilage of Sham and ACLT mice examined by PKR fCLIP-qPCR analysis (n=6). (C) Immunohistochemistry analysis of pPKR, peIF2α, Spi-1 and ADAMTS-5 protein expression in the articular cartilage of Sham and ACLT mice. Quantification of Integrated Optical Density (IOD) is shown on the right (n=6). (D) Spi-1 binding on ADAMTS-5 promoter in the articular cartilage of Sham and ACLT mice examined by ChIP. Quantification is shown below the electrophoretic gel image (n=6). Data (mean ± std) was representative of three independent experiments. An unpaired Student's t-test was conducted to compare two groups. *: $p<0.05$, **: $p<0.01$, ***: $p<0.001$, ns: no significance.

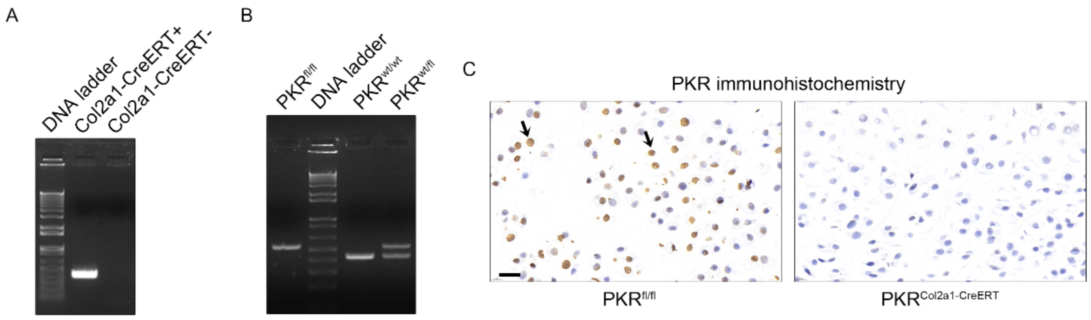

Figure S9

Figure S9: Establishment of PKR conditional knockout mice $PKR^{Col2a1-CreERT}$

(A) Genotyping of Col2a1-CreERT mice. The band of Cre^{+} mice is 358 bp. (B) Genotyping of $PKR^{fl/fl}$ mice. The band of $PKR^{fl/fl}$ mice is 432 bp. The band of $PKR^{fl/wt}$ mice is 432 bp and 348 bp. The band of $PKR^{wt/wt}$ mice is 348 bp. (C) Representative IHC staining of PKR from articular cartilage sections of $PKR^{fl/fl}$ mice and $PKR^{Col2a1-CreERT}$ mice. The arrows point at the positively stained chondrocytes.

Supplementary Table S1: Primer sequences used in RT-qPCR

Gene	Primer sequences (5'-3')
IFNB1	AGTAGGCGACACTGTTCGTG (F)
	GCCTCCCATTCAATTGCCAC (R)
LY6E	ATCTTCTTGCCAGTGCTGCT (F)
	GAACAGGTCTTGCTCAGGCT (R)
STAT1	TCTGTGTCTGAAGTTCACCCTT (F)
	CAGAGCCCACTATCCGAGAC (R)
IRF7	CTGTGGACACCTGTGACACC (F)
	TGCCCTCTCAGGAGCCAA (R)
IFIT3	CAGAGGGCAGTCATGAGTGAGG (F)
	CTTCAGCTTGCCGTAAGCATTC (R)
MX1	CAGCTCAGGGGCTTTGGAAT (F)
	CCTTGGAATGGTGGCTGGAT (R)
IFI27	TCCTTCTTTGGGTCTGGCTG (F)
	TGGCCACAACTCCTCCAATC (R)
ADAMTS-5	GCCTCTCCCATGACGATTCC (F)
	TCGTGGTAGGTCCAGCAAAC (R)
ND1 (Human)	TCAAACTCAAACTACGCCCTG (F)
	CGCAAATGGGCGGTAGGCGTGGTT<br>GTGATAAGGGTGGAGAGG (R)
ND4 (Human)	CTCACACTCATTCTCAACCCC (F)
	CGCAAATGGGCGGTAGGCGTGTGT<br>TTGTCGTAGGCAGATGG (R)
ND5 (Human)	CTAGGCCTTCTTACGAGCC (F)
	CGCAAATGGGCGGTAGGCGTGTTT<br>GGGTTGAGGTGATGATG (R)
ND6 (Human)	TGCTGTGGGTGAAAGAGTATG (F)
	CGCAAATGGGCGGTAGGCGTGCC<br>CATAATCATACAAAGCCCC (R)
CYTB (Human)	CAATTATACCCTAGCCAACCCC (F)
	CGCAAATGGGCGGTAGGCGTGGG<br>ATAGTAATAGGGCAAGGACG (R)
Spi-1	AAAATCAGGAACTTGTGCTGGC (F)
	GGGGAAACCCTTCCATTTTGC (R)
ND1 (Mouse)	TCCGAGCATCTTATCCACGC (F)
	GTATGGTGGTACTCCCGCTG (R)
ND4 (Mouse)	TAATCGCACATGGCCTCACA (F)
	CATTTGAAGTCCTCGGGCCA (R)
ND5 (Mouse)	CAGCACAATTTGGCCTCCAC (F)
	TAGTCGTGAGGGGGTGGAAT (R)
ND6 (Mouse)	CCCGCAAACAAAGATCACCC (F)
	TCTTGATGGTTTGGGAGATTGGT (R)
CYTB (Mouse)	TGCATACGCCATTCTACGCT (F)
	AGGCTTCGTTGCTTTGAGGT (R)
β-tubulin (Human)	GCGCTTATCGAAGTGTGGTC (F)
	ACCCTTCCCCTAGACACTCG (R)
β-tubulin (Mouse)	CGGTGCTAAGTTCTGGGAGG (F)
	CCCAGACTGACCGAAAACGA (R)

F: forward, R: reverse

Supplementary Table 2: Clinical characteristics of specimens and gene expression data

Patient ID	Age	Gender	Diagnosis	Stage	ND1 level (% β-tubulin)				ND4 level (% β-tubulin)			
					value	mean	std	p value	value	mean	std	p value
#1	61	F	Knee OA	1	4.97				7.14			
#2	66	F	Knee OA	1	11.86	8.07	2.87		14.06	11.32	4.69	
#3	66	M	Knee OA	1	8.30				7.56			
#4	69	F	Knee OA	1	7.17				16.51			
#5	70	M	Knee OA	2	10.31				17.27			
#6	71	M	Knee OA	2	25.54	17.65	7.97	0.2054 (Stage 2 vs. Stage 1)	24.54	20.90	3.61	0.0121 (Stage 2 vs. Stage 1)
#7	72	F	Knee OA	2	11.28				18.39			
#8	72	F	Knee OA	2	23.47				23.41			
#9	73	F	Knee OA	3	21.05				20.95			
#10	73	F	Knee OA	3	20.27				14.31			
#11	75	M	Knee OA	3	10.86	21.81	8.80	0.0372 (Stage 3 vs. Stage 1)<br>0.6690 (Stage 3 vs. Stage 2)	19.69	16.24	3.38	0.1618 (Stage 3 vs. Stage 1)<br>0.1901 (Stage 3 vs. Stage 2)
#12	76	F	Knee OA	3	36.27				13.04			
#13	76	F	Knee OA	3	15.97				13.22			
#14	80	F	Knee OA	3	26.43				16.23			

Patient ID	ND5 level (% β-tubulin)				ND6 level (% β-tubulin)				CYTB level (% β-tubulin)			
	value	mean	std	p value	value	mean	std	p value	value	mean	std	p value
#1	2.38				6.78				8.36			
#2	16.86	12.79	7.83		2.46	3.96	1.95		14.19	15.86	8.23	
#3	20.37				2.91				27.59			
#4	11.54				3.68				13.30			
#5	8.59				11.32				41.17			
#6	9.87	22.33	15.56	0.4524 (Stage 2 vs. Stage 1)	10.98	14.12	3.44	0.1392 (Stage 2 vs. Stage 1)	69.52	47.40	17.12	0.0617 (Stage 2 vs. Stage 1)
#7	30.99				16.78				28.85			
#8	39.86				17.40				50.05			
#9	23.28				26.70				66.45			
#10	38.38				13.97				62.91			
#11	25.89	25.90	8.71	0.1915 (Stage 3 vs. Stage 1)<br>0.8675 (Stage 3 vs. Stage 2)	24.97	26.82	9.77	0.0009 (Stage 3 vs. Stage 1)<br>0.0387 (Stage 3 vs. Stage 2)	37.94	54.11	21.04	0.0145 (Stage 3 vs. Stage 1)<br>0.8227 (Stage 3 vs. Stage 2)
#12	28.62				20.03				85.43			
#13	27.69				33.84				42.38			
#14	11.54				41.43				29.54			

Patient ID	pPKR protein (% β-tubulin)				peIF2α protein (% β-tubulin)				ADAMTS-5 mRNA (% β-tubulin)			
	value	mean	std	p value	value	mean	std	p value	value	mean	std	p value
#1	10.61				4.85				6.04			
#2	10.36	10.99	0.60		5.56	4.90	0.45		12.62	11.78	4.95	
#3	11.63				4.58				17.96			
#4	11.36				4.62				10.52			
#5	24.01				8.82				17.86			
#6	16.23	21.24	5.77	0.0245 (Stage 2 vs. Stage 1)	7.55	9.38	1.71	0.0230 (Stage 2 vs. Stage 1)	27.05	19.85	6.00	0.7458 (Stage 2 vs. Stage 1)
#7	16.68				11.65				12.85			
#8	28.05				9.50				21.63			
#9	22.67				17.25				69.48			
#10	26.76				11.47				92.79			
#11	30.90	28.91	5.24	0.0003 (Stage 3 vs. Stage 1)<br>0.0642 (Stage 3 vs. Stage 2)	17.53	14.07	2.65	<0.0001 (Stage 3 vs. Stage 1)<br>0.0103 (Stage 3 vs. Stage 2)	43.34	58.39	22.06	0.0018 (Stage 3 vs. Stage 1)<br>0.0067 (Stage 3 vs. Stage 2)
#12	29.02				13.33				53.40			
#13	37.97				12.75				62.26			
#14	26.16				12.12				29.07			

F:female, M: male, OA: osteoarthritis, std: standard deviation
