# Supplementary figures and images for: Mitochondrial dysfunction activates ADAMTS-5 expression via mt-dsRNA-PKR-Spi-1 axis in osteoarthritic chondrocytes
Source: iScience. 2026 May 20;29(6):115980. doi: 10.1016/j.isci.2026.115980 (PMC13214264; doi:10.1016/j.isci.2026.115980)

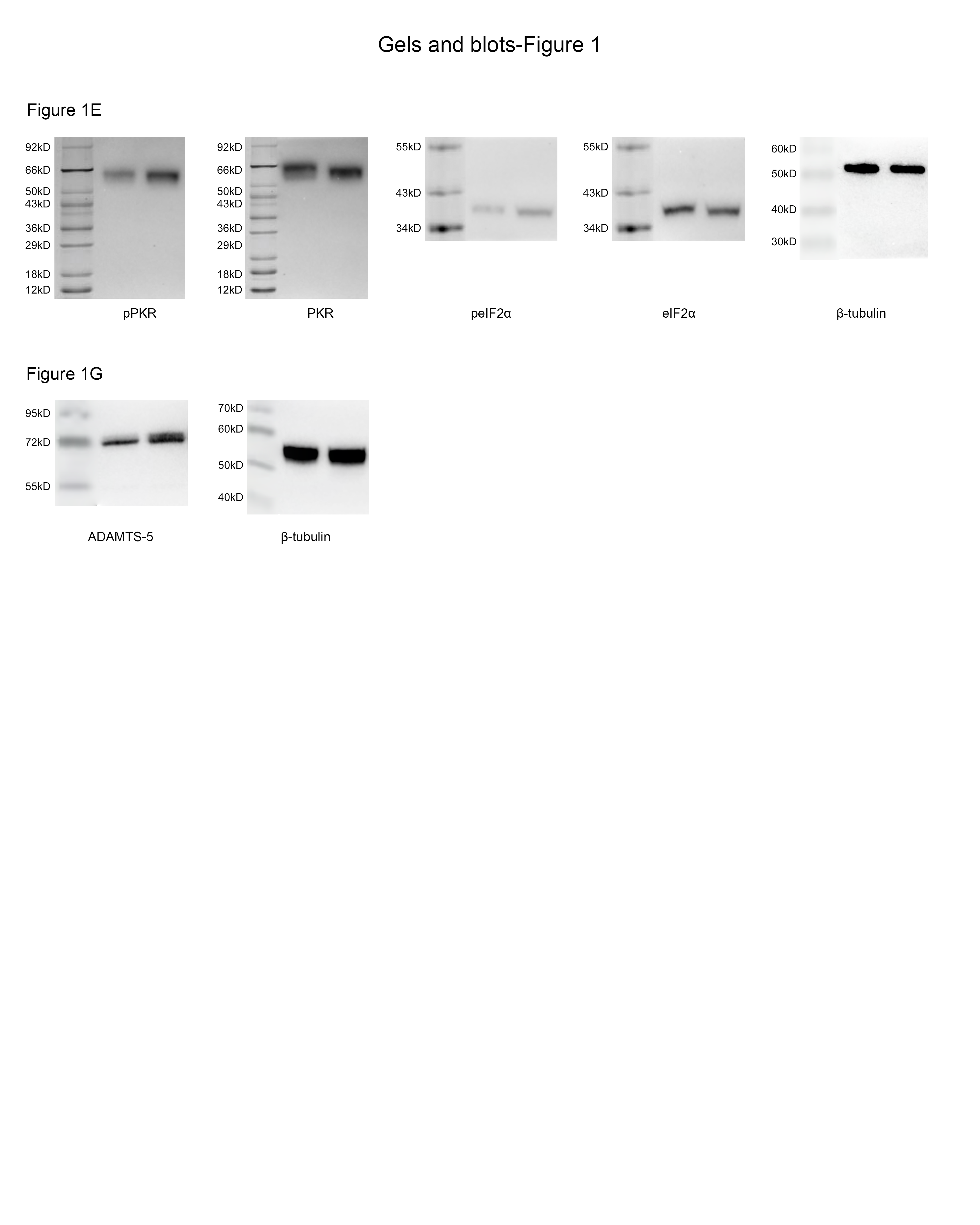

Supplement: Document S2. Gels and Blots [file mmc3.zip › Gels and blots-Figure 1.tif]

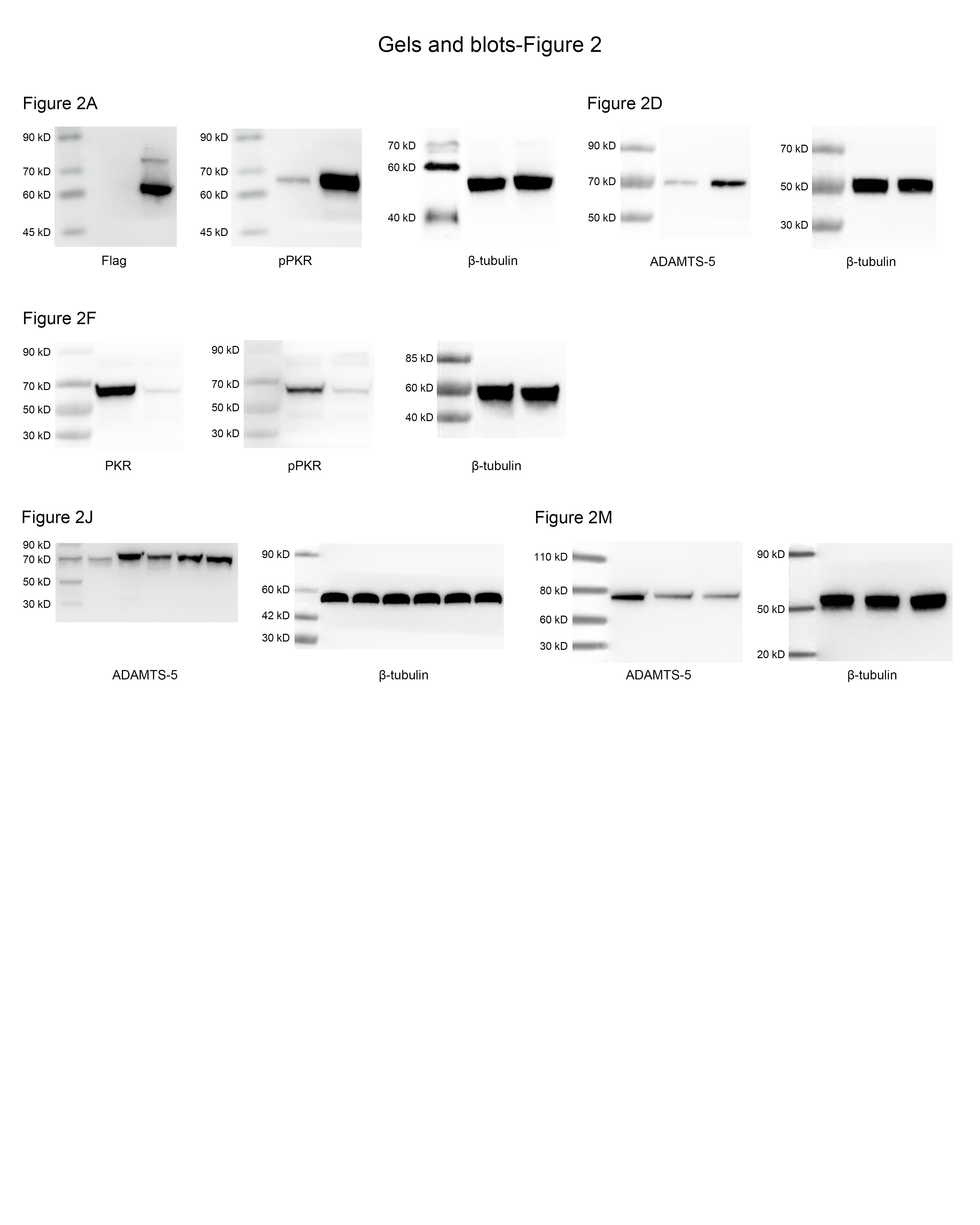

Supplement: Document S2. Gels and Blots [file mmc3.zip › Gels and blots-Figure 2.tif]

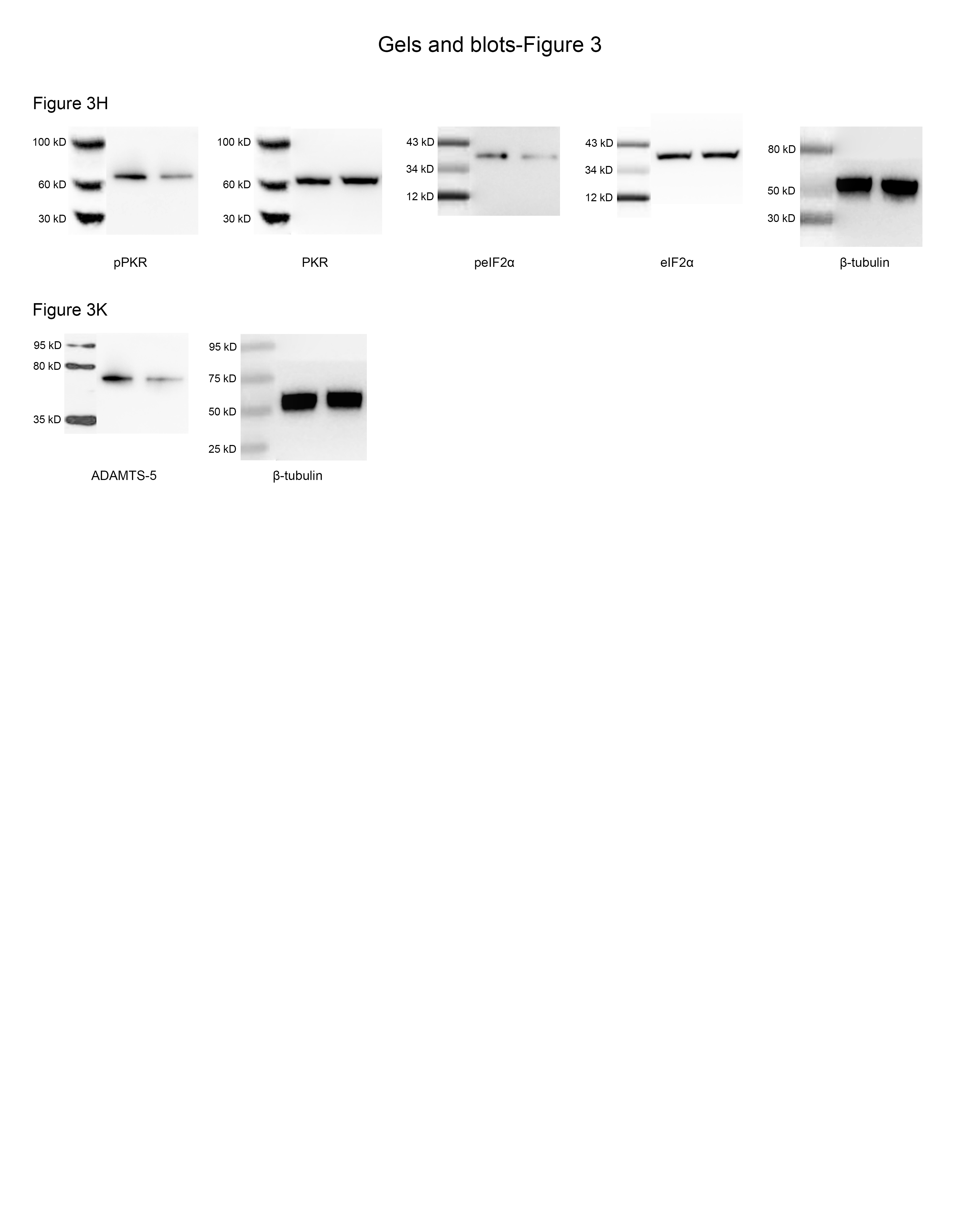

Supplement: Document S2. Gels and Blots [file mmc3.zip › Gels and blots-Figure 3.tif]

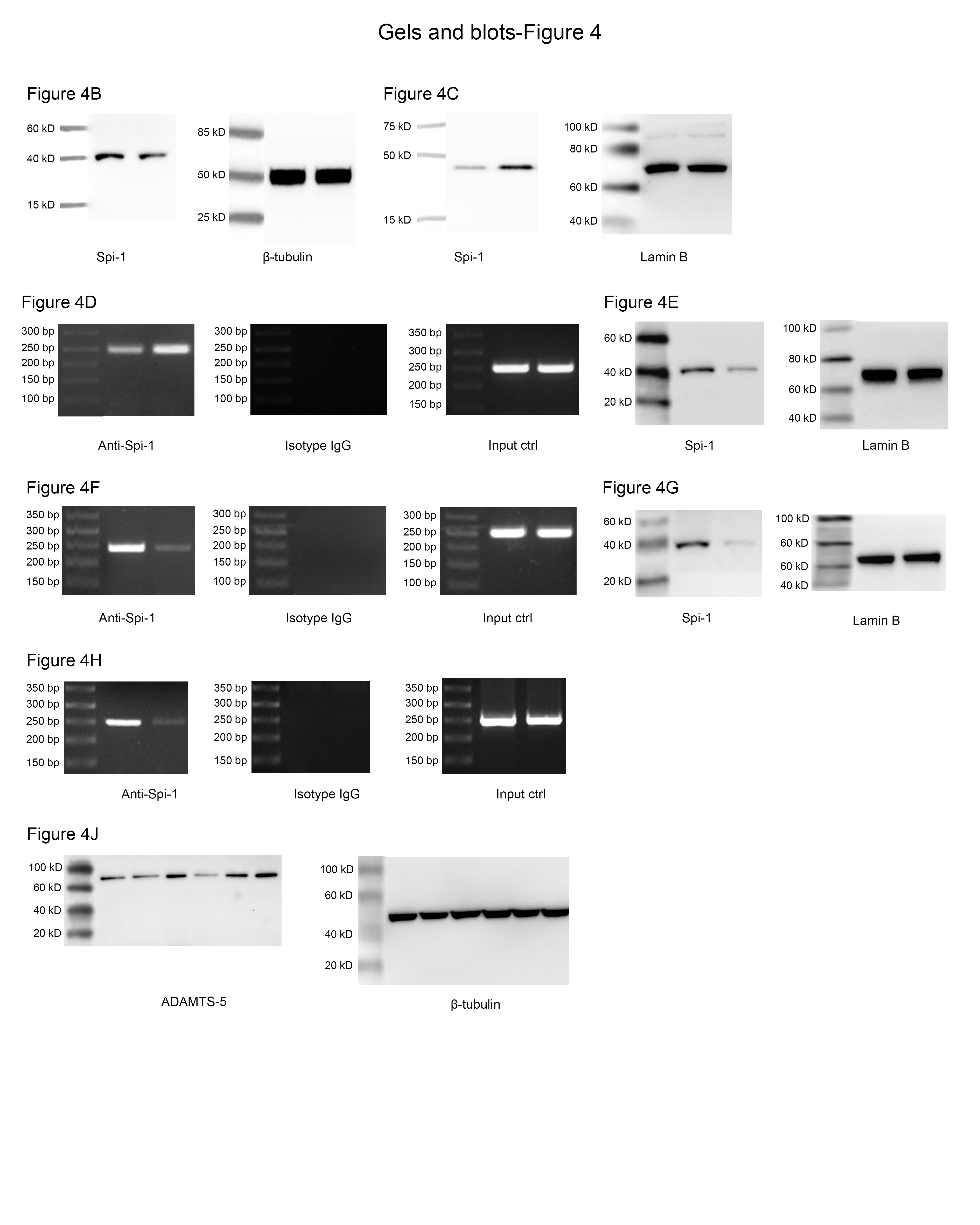

Supplement: Document S2. Gels and Blots [file mmc3.zip › Gels and blots-Figure 4.tif]

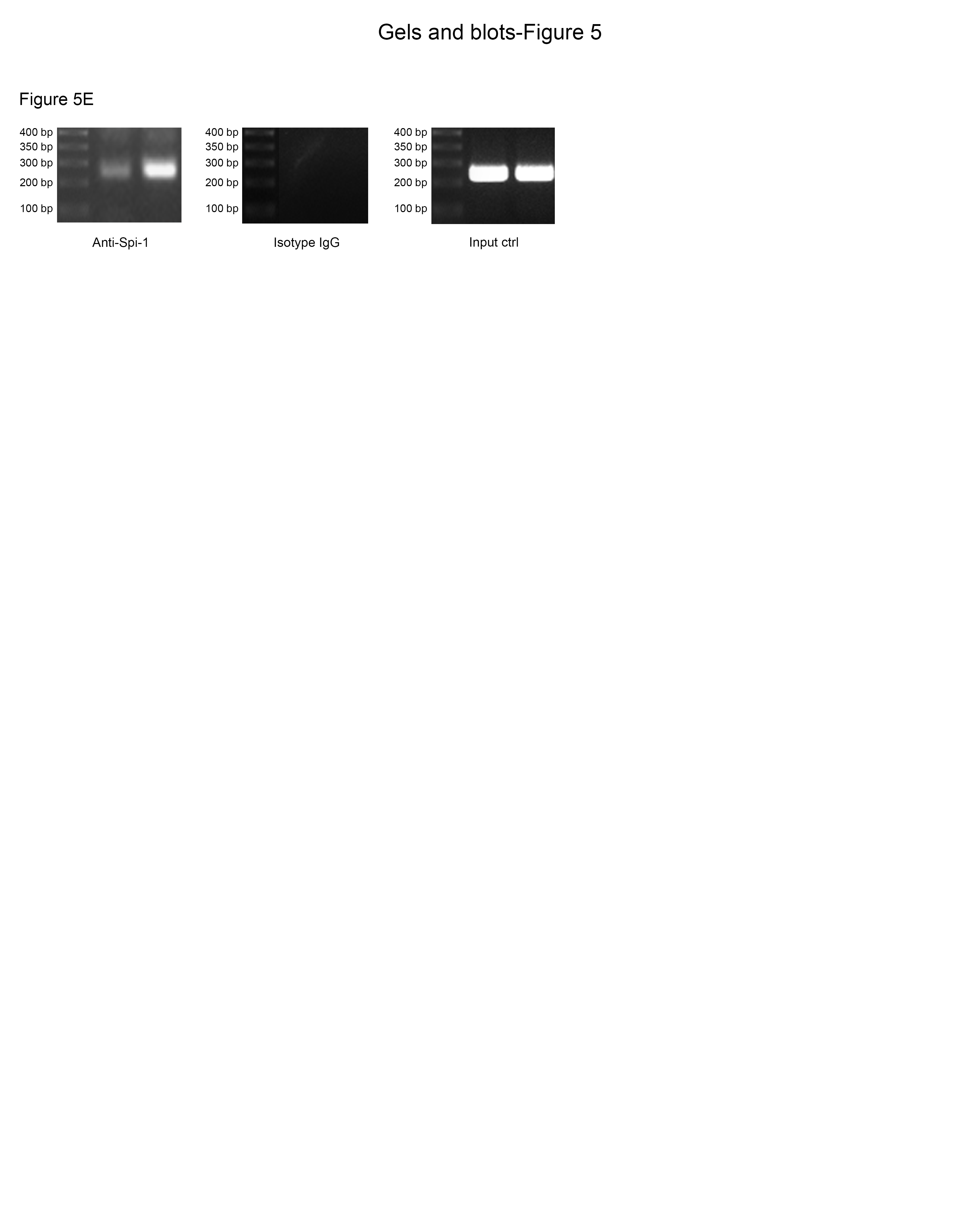

Supplement: Document S2. Gels and Blots [file mmc3.zip › Gels and blots-Figure 5.tif]

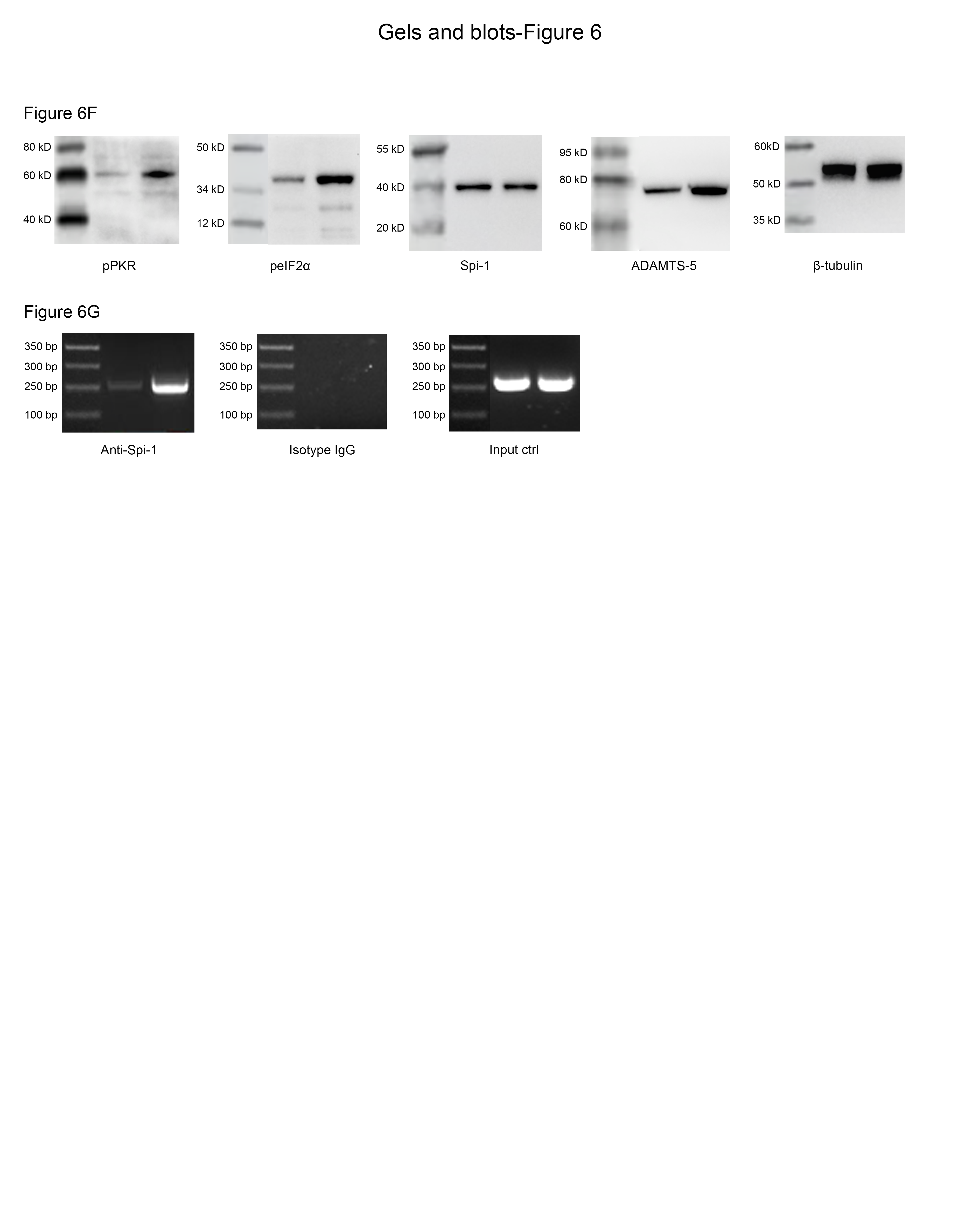

Supplement: Document S2. Gels and Blots [file mmc3.zip › Gels and blots-Figure 6.tif]

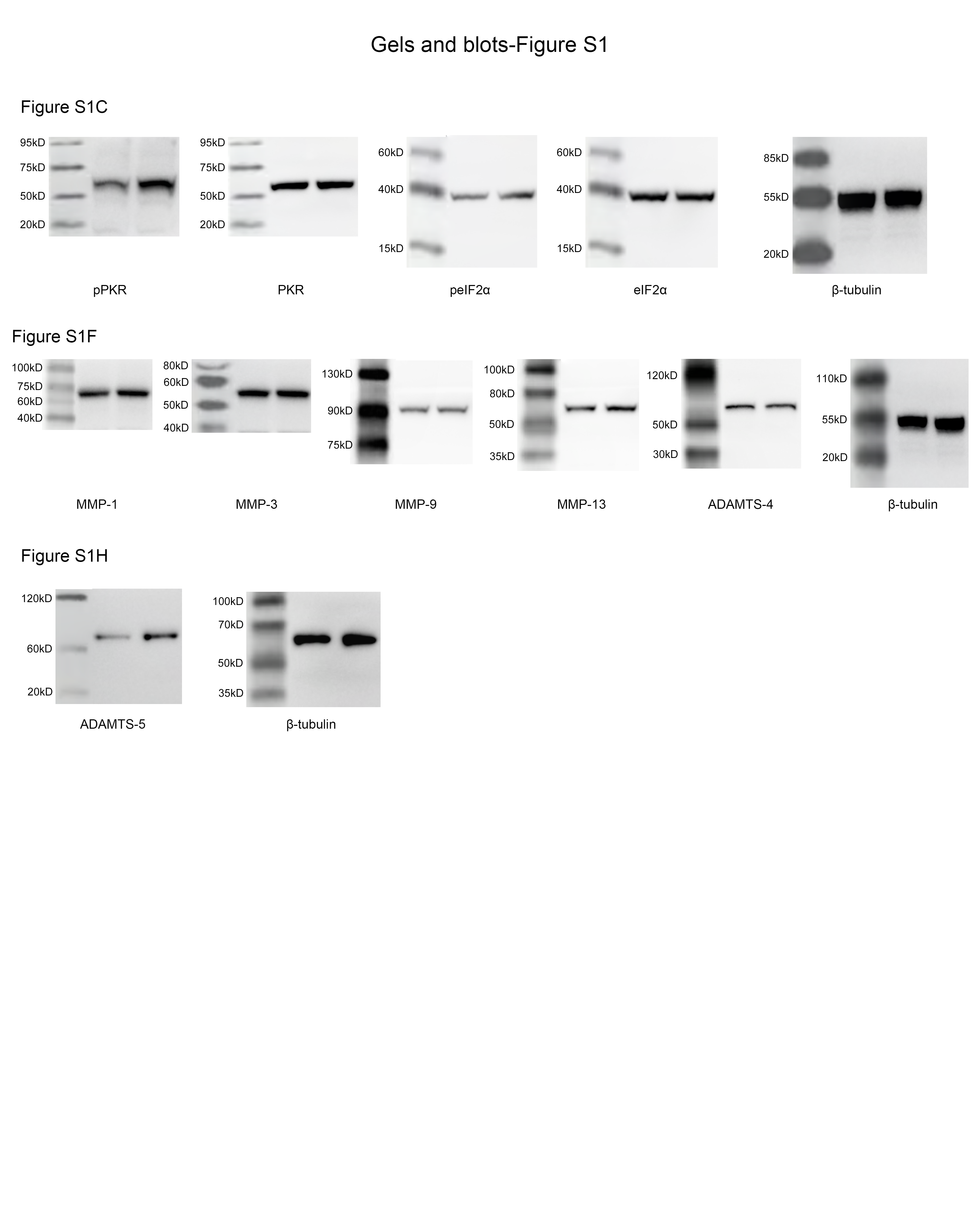

Supplement: Document S2. Gels and Blots [file mmc3.zip › Gels and blots-Figure S1.tif]

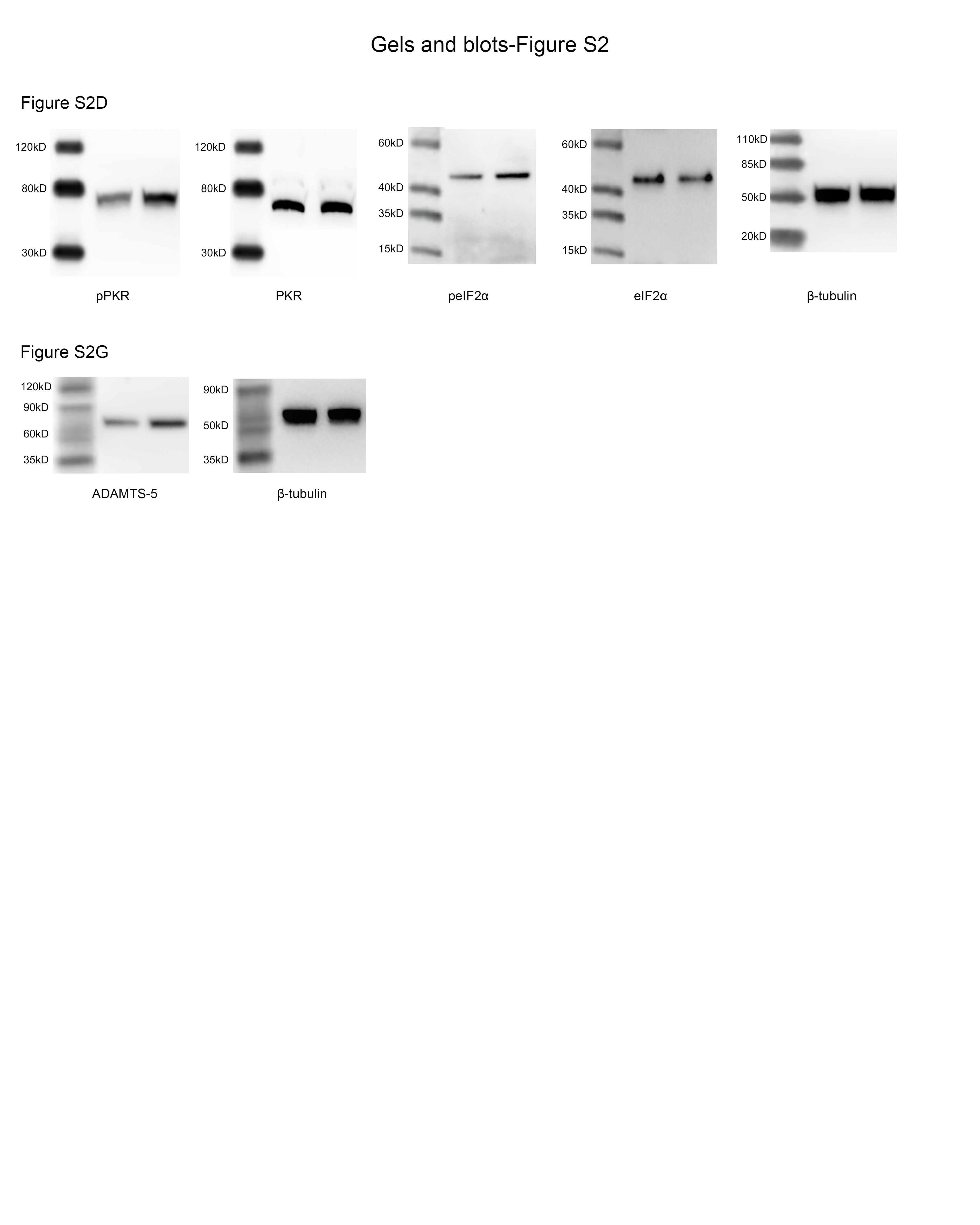

Supplement: Document S2. Gels and Blots [file mmc3.zip › Gels and blots-Figure S2.tif]

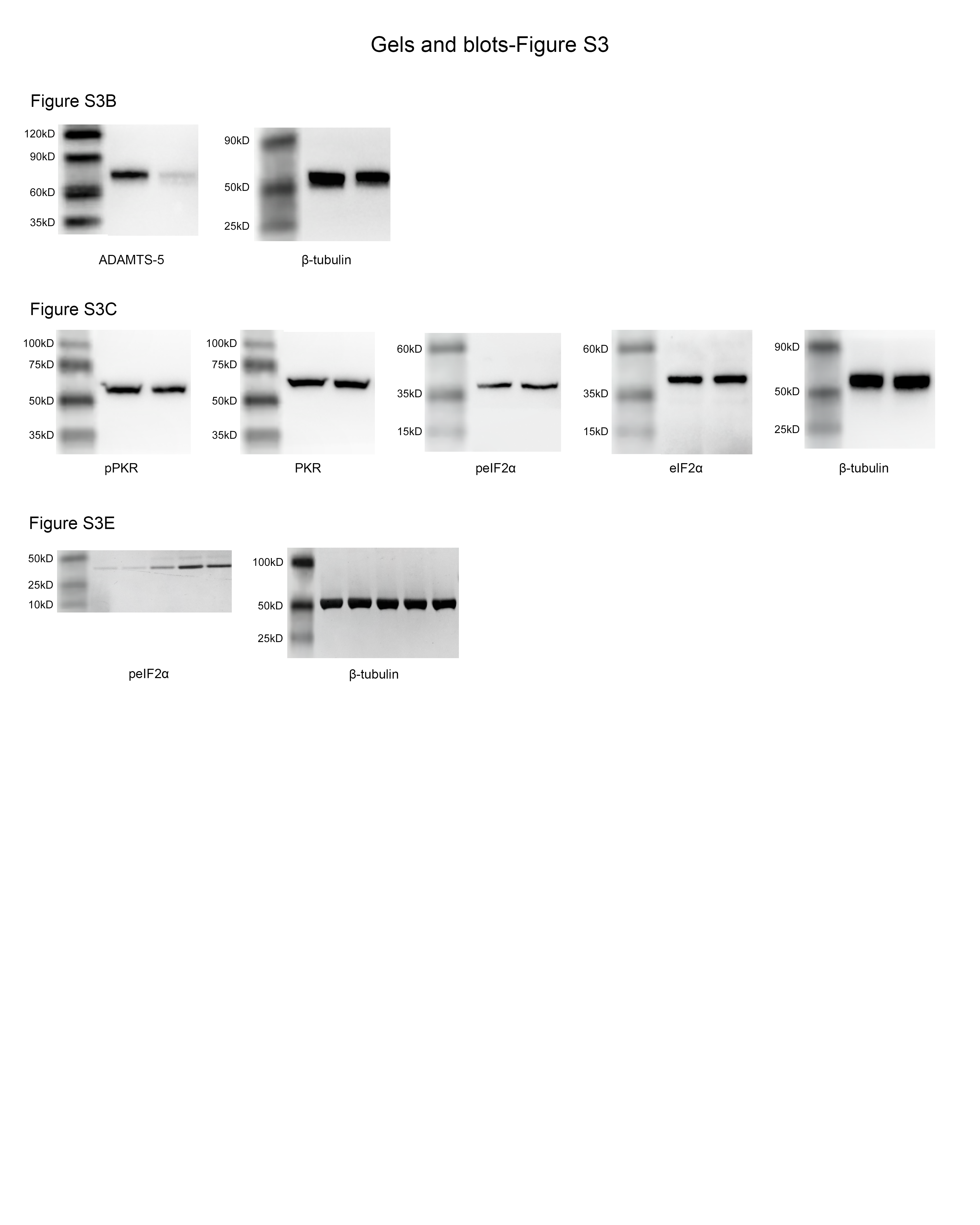

Supplement: Document S2. Gels and Blots [file mmc3.zip › Gels and blots-Figure S3.tif]

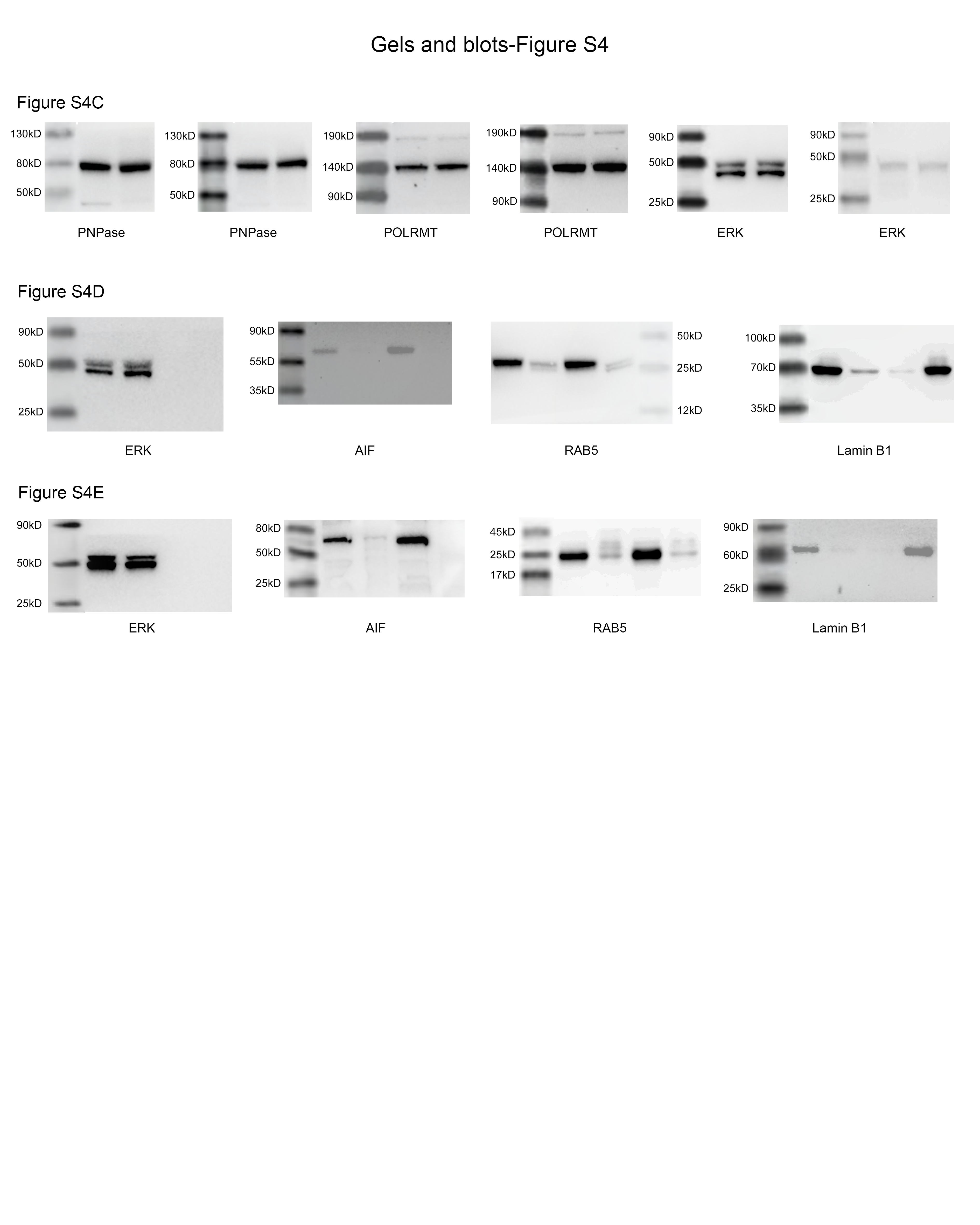

Supplement: Document S2. Gels and Blots [file mmc3.zip › Gels and blots-Figure S4.tif]

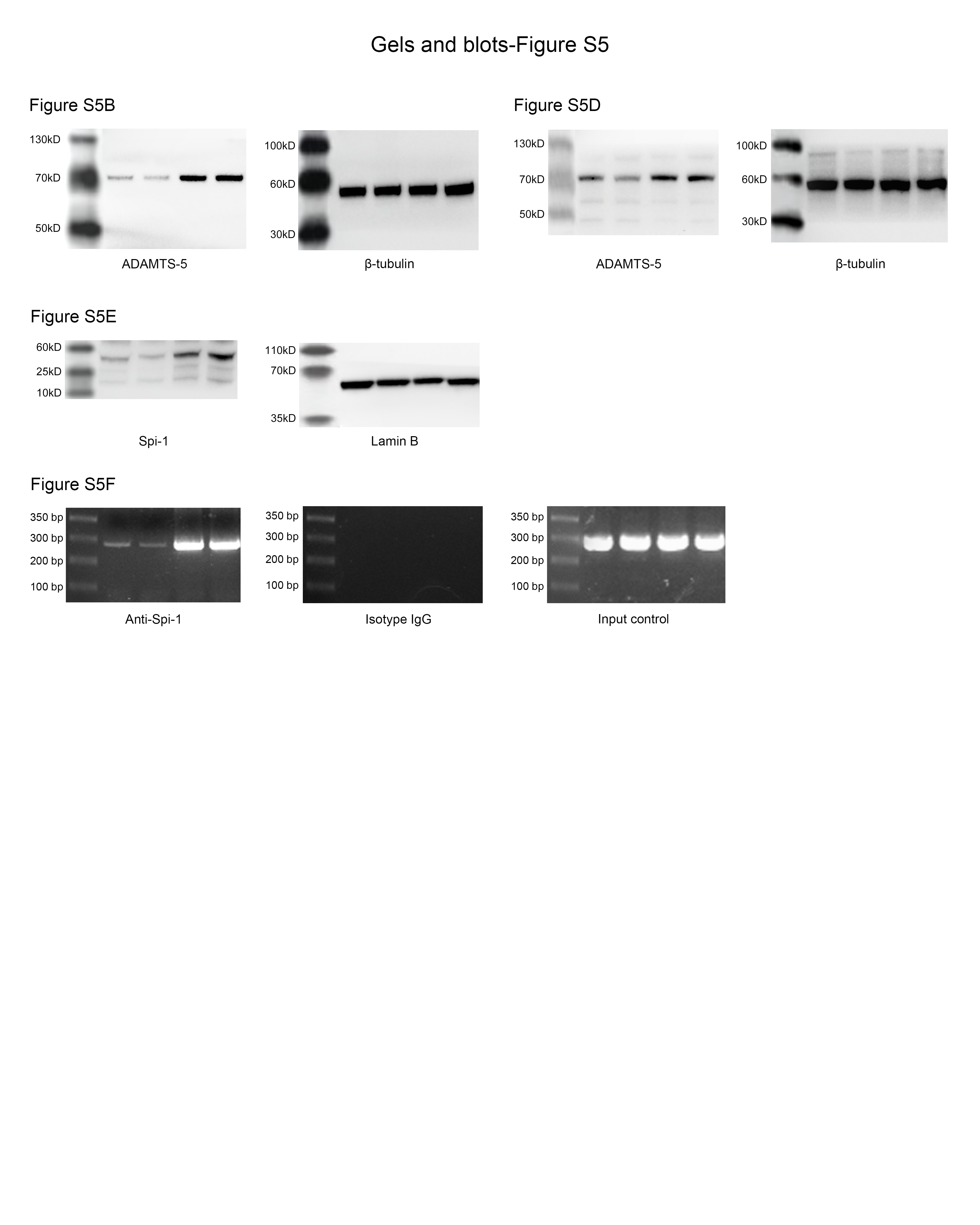

Supplement: Document S2. Gels and Blots [file mmc3.zip › Gels and blots-Figure S5.tif]

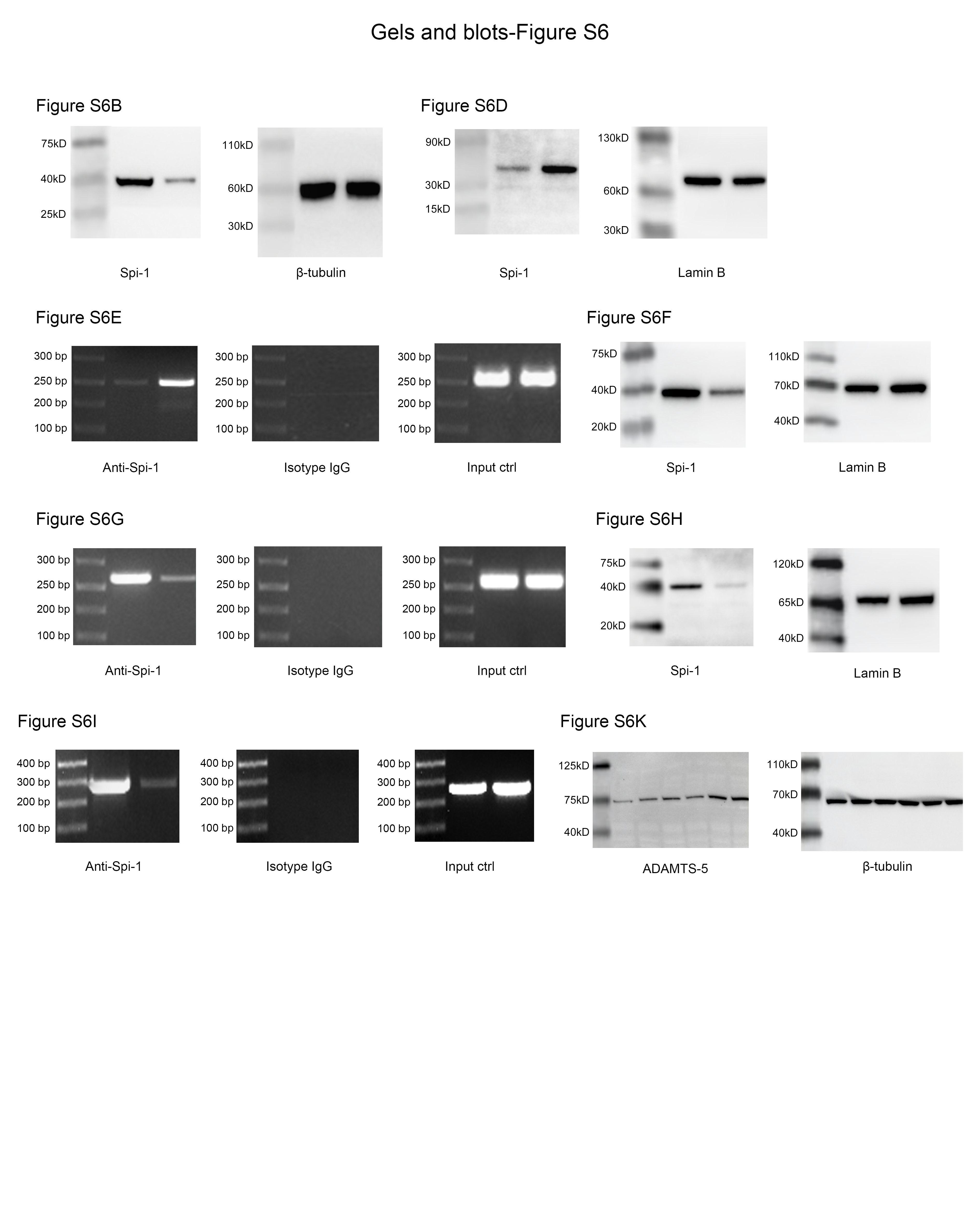

Supplement: Document S2. Gels and Blots [file mmc3.zip › Gels and blots-Figure S6.tif]

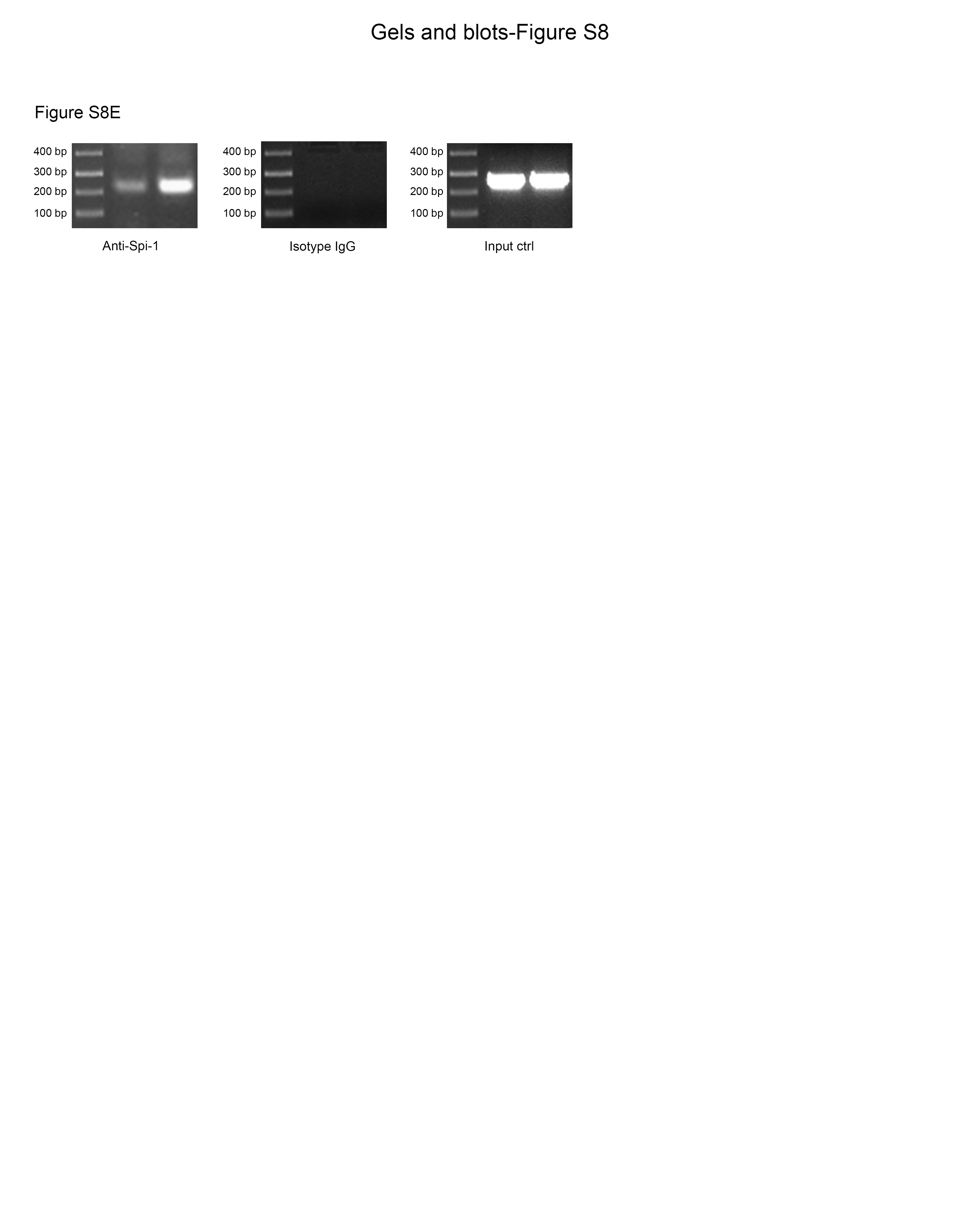

Supplement: Document S2. Gels and Blots [file mmc3.zip › Gels and blots-Figure S8.tif]
